# Mapping the Evidence for Oropouche Virus to Inform Research Prioritisation

**DOI:** 10.1002/rmv.70222

**Published:** 2026-10-24

**Authors:** Sowsan F. Atabani, Matthew Bosworth, Amy Bobbins, Beti Evans, Zalaya Simmons, Nicola Pearce‐Smith, Julia A. Tree, Charles Dearman, Yper Hall, Daphne Duval

**Affiliations:** ^1^ UK Health Security Agency (UKHSA) Southwest Regional Laboratory and Severn Infection Services Bristol NHS Foundation Trust Bristol UK; ^2^ Evidence and Knowledge Division Knowledge and Library Services, Research UKHSA London UK; ^3^ Centre for Guidelines National Institute for Health and Care Excellence London UK; ^4^ Centre for Climate and Health Security CSO Group, UKHSA London UK; ^5^ Porton Down Salisbury Wiltshire UK; ^6^ CSO Private Office, UKHSA London UK; ^7^ Southend‐on‐Sea City Council Southend‐on‐Sea Essex UK

**Keywords:** evidence gap maps, mapping review, oropouche virus, priority pathogen, rapid systematic methods, research prioritisation

## Abstract

Oropouche virus (OROV) infection has gained increasing public health attention due to the ongoing outbreaks in South and Central America. The World Health Organisation (WHO) lists OROV as a priority pathogen for epidemic and pandemic preparedness. This work aimed to identify and categorise the available evidence on OROV to inform research prioritisation. We conducted a rapid mapping review (search date: up to 5 June 2025) to identify primary studies on OROV and categorised these by area of research, study design, and geographic region using a pre‐defined framework. Included studies were displayed in an interactive evidence gap map (EGM) to identify any evidence gaps. In total, 269 primary studies on OROV were included and mapped into the EGM. Of these, the majority of data and findings were reported from South America, Central America and the Caribbean, reflecting the geographical epidemiology of OROV. The majority of studies were observational and descriptive, based on specific outbreaks. Limited evidence was found on vector surveillance, viral pathogenesis, diagnostics and immune correlates of protection. No experimental studies on potential therapeutics or vaccines conducted in humans were identified. The findings from our mapping review and EGM highlight the utility of this methodology to rapidly identify gaps in scientific evidence for emerging or re‐emerging pathogens with outbreak potential. The EGM can be used solely, or in conjunction with other prioritisation tools, in order to focus investment and research efforts towards priority topics.

## 1 Introduction

The Peribunyaviridae viral family, in which Oropouche virus (OROV) is a member, has been included in the 2024 World Health Organisation (WHO) Research and Development (R&D) Blueprint for Epidemics and Pandemics global pathogen prioritisation list, as well as in the 2025 UK Health Security Agency (UKHSA) Priority Pathogens R&D tool. These lists aim to identify pathogen families with epidemic and pandemic potential. To that effect, and in collaboration with the WHO, a collaborative open research consortium (CORC) for Peribunyaviridae was established, with the UKHSA as the designated coordinator lead to co‐ordinate and galvanise a global scientific, evidence‐based roadmap for the research into, and development of, countermeasures for OROV infection.

Although initially discovered in 1955, OROV has recently garnered increasing public health attention and concern due to the ongoing and expanding outbreaks in South and Central America and the Caribbean [1]. OROV, a negative sense, tri‐segmented genomic RNA virus was historically associated with epidemic activity near forested regions but is increasingly being reported in urban settings [2]. Transmission has classically been attributed to the biting midge *Culicoides paraensis*, with some, more recent evidence for potential mosquito involvement, supporting both sylvatic and urban cycles of transmission. These shifts have broadened the ecological range of OROV and coincided with detections in areas far from traditional forest habitats [1]. Besides vector‐borne transmission, OROV RNA has been detected in semen, raising the possibility of sexual transmission [3].

Clinical OROV infection can present with a broad‐spectrum of disease severity [4]. From asymptomatic or a non‐specific febrile illness with spontaneous recovery with no severe long‐term sequelae, to neurological complications, ranging from aseptic meningoencephalitis [5] to a possible association with Guillain‐Barre syndrome [6]. Due to the overlapping clinical symptoms and signs of fever, headache and arthralgia, it can be mis‐diagnosed for infection caused by dengue, chikungunya, Zika or malaria, hampering surveillance efforts. Interestingly, OROV infections can follow a biphasic course with recurrence of symptoms approximately 1 week after initial symptom onset [7]. More recently, reports of vertical transmission have been described, associated with miscarriages, stillbirths, and congenital malformations such as microcephaly [8].

In 2024, 19 imported OROV cases in Spain (12 cases), Italy (5 cases), and Germany (2 cases) were reported and 18 cases were associated with travel to Cuba thus highlighting travel associated risk [9]. Although C. *paraensis* is absent in Europe, *Aedes* albopictus mosquitoes can exhibit low vector competence under warm conditions ex vivo, suggesting a theoretical potential for seasonal transmission in parts of southern Europe if epidemiological and climatic conditions converge [10].

According to updates from the Pan American Health Organisation (PAHO) and WHO, as of 14th August 2025, over 12,786 confirmed OROV cases, including four deaths in 2025 have been confirmed. The geographic spread of this disease, seen in more than 11 countries in 2025 [11] highlights urgent needs to fully understand transmission, vectors, diagnostics, surveillance capacity, and clinical impacts, including reported vertical transmission.

In this regard, we conducted a rapid mapping review with an evidence gap map (EGM) to identify and categorise the available evidence on OROV to inform research prioritisation. EGMs are based on systematic methodologies and act as visualisation tools to identify where there is published evidence, where evidence is limited and where there is a complete lack of evidence (‘evidence gaps’). For the EGM, we represented the evidence identified on OROV by area of research, study design and geographic region. This paper summarises the main findings from our rapid mapping review (full report published on the UK government website [12]) and helps to shape priority questions to guide preparedness and response.

## 2 Methods

We used streamlined systematic methods [13] to conduct a rapid mapping review to address the review question ‘what evidence is available on Oropouche virus?’. We followed the PRISMA checklist for scoping reviews [14], which is also applicable to mapping reviews [15].

We produced a protocol before the literature search began. The protocol included the framework for the EGM, which was developed with input from a range of topic experts (including in methodology review, virology and entomology). It was agreed at the protocol stage that critical appraisal would not be undertaken, as the aim of our study was to identify and categorise the available primary studies to inform research prioritisation, regardless of their methodological quality [25]. The protocol is available in Supporting Information S1: material 1.

### 2.1 Eligibility Criteria

We included all primary studies that reported on OROV, including human and animal studies, studies in laboratory settings, and modelling studies. As the aim of our study was to identify primary studies to inform research prioritisation, guidelines, opinion pieces and reviews (systematic or narrative) were excluded. However, the reference lists of relevant reviews were screened as an additional source of evidence (see Search strategy section below). There were no limitations on settings, countries, publication date, or language.

Primary studies published in peer‐reviewed journals, as preprints, or as grey literature were all considered for inclusion (except for conference abstracts, theses, and books or book chapters, which were excluded).

The inclusion and exclusion criteria for this review are available in Table S1 of Supporting Information S1: material 1.

### 2.2 Search Strategy

We conducted searches from database inception on 5 June 2025 in seven databases (Ovid Medline ALL, Ovid Embase, Ovid Global Health, Web of Science Core Collection, medRxiv, bioRxiv, and LILACS Plus). The search strategies were draughted by an information scientist and peer‐reviewed by a second information scientist and are available in Supporting Information S1: material 2.

We also searched seven sources of grey literature on 5 June 2025: the PAHO Institutional Repository for Information Sharing (IRIS) [16], the Caribbean Public Health Agency (CARPHA) evidence portal [17], Redalyc [18], and websites from the WHO, Centres for Disease Control and Prevention (CDC), PAHO and European Centre for Disease Prevention and Control (ECDC).

Additional sources of evidence were identified by consulting with experts from the *Bunyaviricetes* CORC and screening lists of relevant systematic and mapping reviews identified during the searches [4, 19, 20, 21, 22, 23]. Of note, one of the reference lists that we screened was an EGM hosted on the PAHO dashboard [31].

### 2.3 Screening

We screened the search results (databases and grey literature) using EPPI‐Reviewer web version 4 (EPPI‐R) [24]. Title and abstract screening was completed in duplicate by two reviewers for 20% of the records and the remainder were screened by one reviewer. The machine learning assisted screening system in EPPI‐Reviewer was used to screen the database search results in order of likely relevance.

For full text screening, 5% of the records included on title and abstract were screened in duplicate by two reviewers in a pilot phase. The remainder were screened on full text by one reviewer and checked by a second.

For the additional sources of evidence (reference lists of relevant systematic and mapping reviews and expert consultation), the screening was performed in one step by one reviewer.

### 2.4 Data Charting

We coded the study designs, areas of research and geographic regions of the included studies in EPPI‐R. Data extraction was limited to the data needed for the map, using a pre‐defined framework (see protocol in Supporting Information S1: material 1).

Coding was initially done in duplicate by two reviewers for 10% of the included studies and confirmed with a virologist, with the remainder conducted by one reviewer and checked by a second. Discrepancies were resolved by discussion with a third reviewer and/or a virologist when needed.

### 2.5 Visual Synthesis

We used EPPI‐Mapper [26] to create an interactive EGM using the coding extracted to visualise the evidence identified on OROV by area of research, study design, and geographic region.

During the preparation of this work the authors used M365 Co‐pilot for language editing. After using this tool/service, the authors reviewed and edited the content as needed and take full responsibility for the content of the published article.

## 3 Results

### 3.1 Search Results

The database searches returned 3796 records, of which 236 met the inclusion criteria. A total of 599 records were identified from other methods (grey literature searches, searching of reference lists of relevant systematic or mapping reviews, and consultation with CORC members), of which 33 were unique and met the inclusion criteria.

In total, 269 studies were included in this review. The list of included studies is available in Supporting Information S1: material 3. The PRISMA diagram is shown in Figure 1. The list of the 332 records excluded on full text can be found in Supporting Information S1: material 4.

**FIGURE 1:**
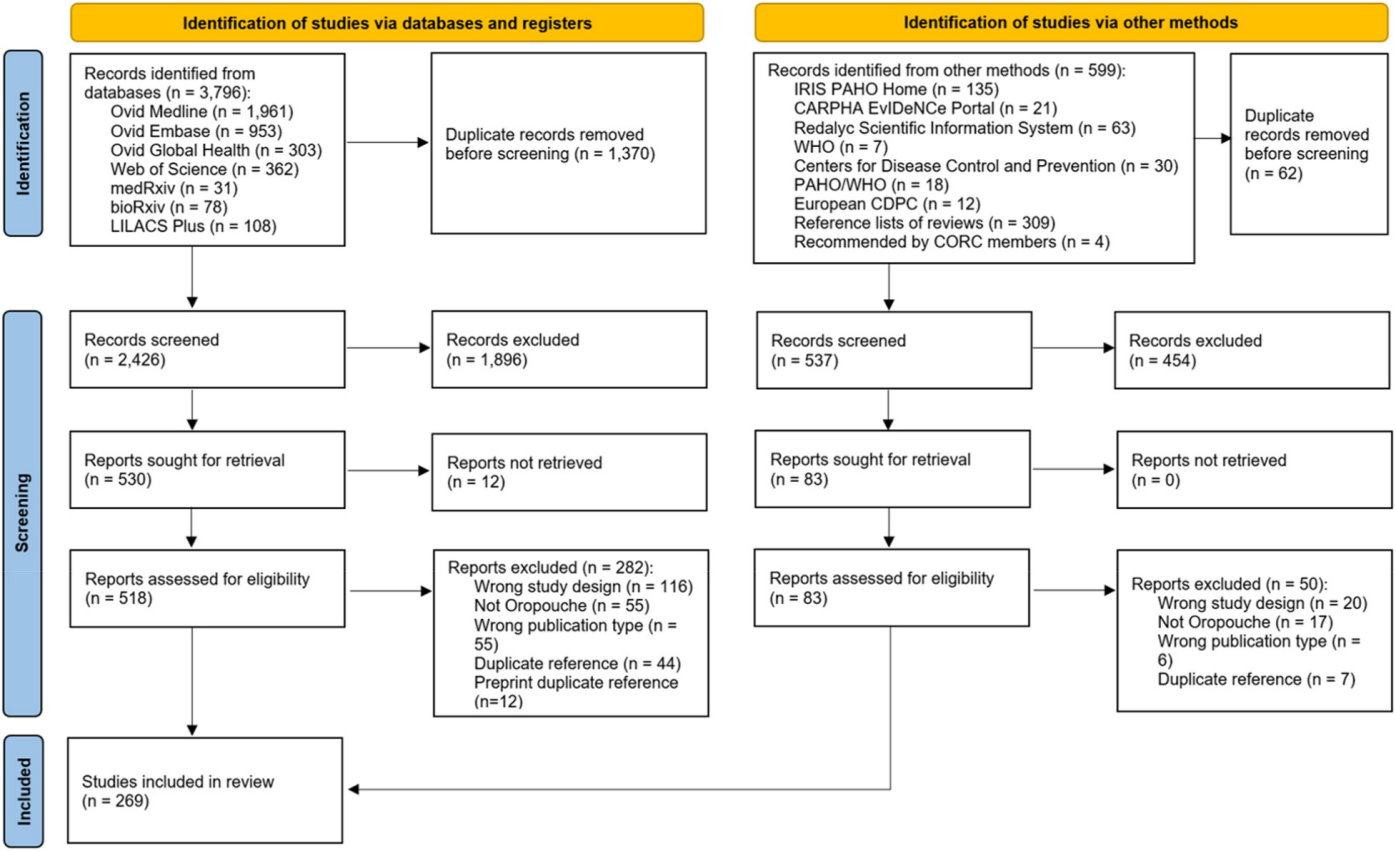
PRISMA diagram.

### 3.2 Study Characteristics

Characteristics of the studies identified in terms of study designs, areas of research and geographic regions (data charting) are provided in Supporting Information S2: material 5.

Of the 269 studies, 122 were observational studies (mainly cross‐sectional and descriptive studies), 74 were laboratory studies, 20 were zoonotic field studies, and 11 were modelling studies. The remaining 42 studies had multiple designs (e.g., a paper that reported on an observational study in humans as well as on a laboratory study or zoonotic field study). No interventional studies conducted in humans were identified.

In terms of areas of research, 152 of the 269 studies reported on epidemiology and surveillance in humans, 92 on virology and pathogenesis, 57 on vector and ecology, 25 on diagnostics, 14 on medical countermeasures (MCM) and only one study on public health and social measures. Some of the studies reported on more than one area of research.

Of the 269 studies, 233 reported data and findings from South America (205 studies) or Central America and the Caribbean (28 studies), reflecting the geographical epidemiology of OROV. In addition, 32 studies reported data and findings from Europe, 28 from North America, and 3 from Asia and Oceania (some studies reported on multiple geographic areas). No studies from Africa were identified.

### 3.3 Visual Synthesis

The 269 studies identified were mapped by area of research, study design, and geographic region into an interactive EGM available online [27]. The map is interactive and categories can be expanded by clicking on the double arrows. A screenshot of the map is presented in Figure 2.

**FIGURE 2:**
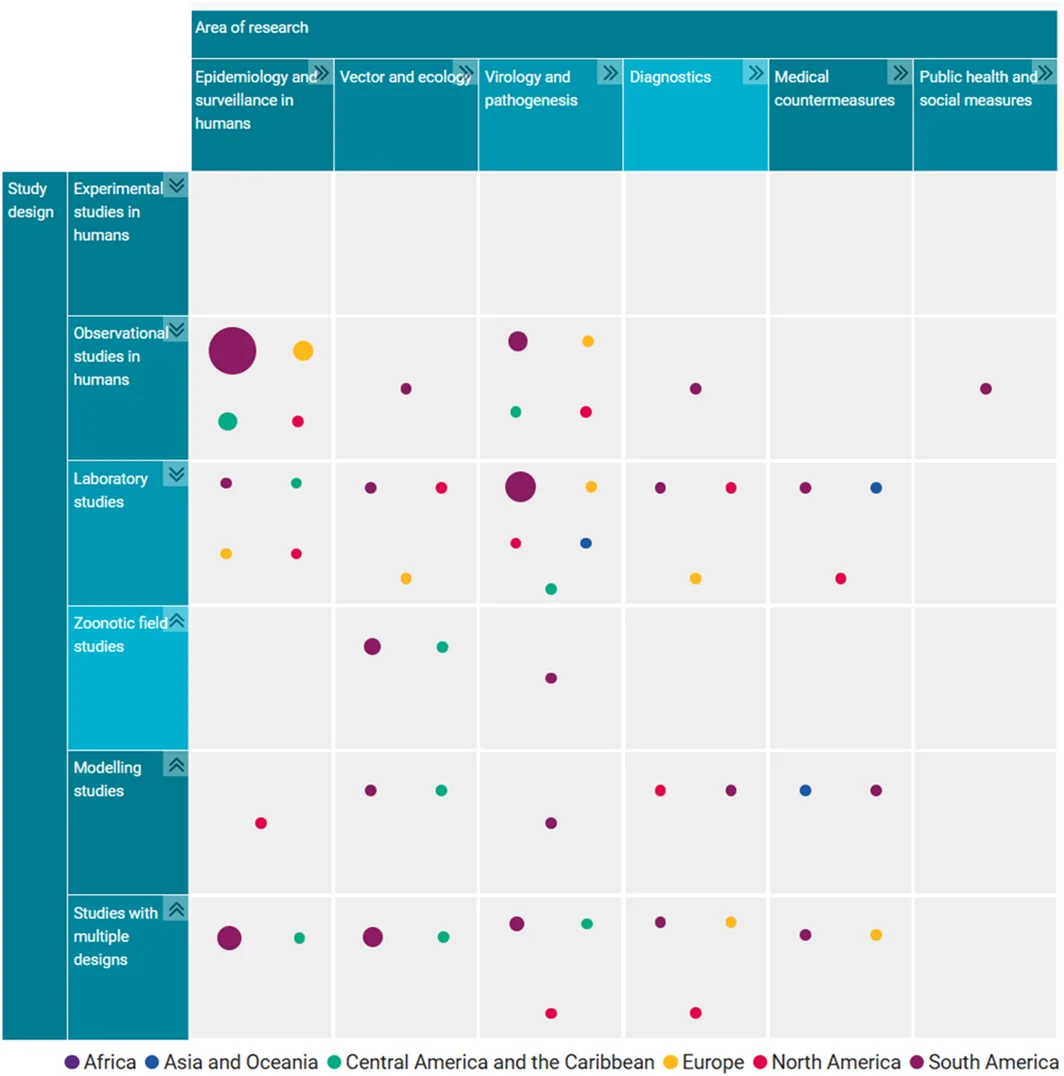
Screenshot of the EGM displaying the studies identified by area of research, study design and geographic region. The size of the bubble represents the number of studies identified for each geographic region by range rather than being directly proportional; in this figure, the smallest bubble size represents up to 10 studies, and the largest bubble represents 95 studies.

### 3.4 Synthesis by Area of Research

In the following, we provide an overview of the evidence identified by area of research. Further details are available in the corresponding report [12] and interactive EGM [27]. Some studies reported on more than one area of research and/or more than one geographic region.

#### 3.4.1 Epidemiology and Surveillance in Humans

The epidemiology and surveillance of OROV in humans was the area of research for which the most evidence was identified (152 out of 269 studies). Of these, 125 reported on ‘surveillance in humans’ and 70 on ‘natural history and clinical manifestations’. The evidence was more limited on ‘modes of transmission’ with only seven studies identified. One study reported on the potential spread of OROV by air travel.

Of the 152 studies, 119 were observational studies, including 46 cross‐sectional studies and 61 descriptive studies.

#### 3.4.2 Virology and Pathogenesis

Ninety‐two studies reported on virology and pathogenesis, of which 62 reported on ‘viral genomics’, 11 on ‘host immune responses’, 10 on ‘viral replicative mechanisms’, 9 on ‘virus tropism’ and 5 on other topics related to virology and pathogenesis. Of the 92 studies, 42 were laboratory studies (including 20 genome sequencing studies and 15 in‐vitro studies) and 24 were observational studies (including 11 cross‐sectional studies and 10 descriptive studies).

#### 3.4.3 Vector and Ecology

Fifty‐seven studies reported on vector and ecology, of which 25 reported on ‘surveillance in animal reservoirs’, 22 on ‘surveillance in arthropod vectors’, 12 on ‘climate and environmental factors’ and 10 on ‘vector competence’. The evidence was mainly based on studies which combined different study designs (21 studies) and zoonotic field studies (20 studies).

#### 3.4.4 Diagnostics

Twenty‐five studies reported on OROV diagnostics, of which 12 reported on ‘molecular diagnostics’, 4 on ‘viral sequencing’, 4 on ‘serological diagnostics’, 2 on ‘clinical diagnostics’, one on ‘assay standards’ and 4 on other types of diagnostics. The evidence was mainly based on laboratory studies (7 in vitro studies, 4 genome sequencing studies and 7 other laboratory studies).

#### 3.4.5 Medical Countermeasures

Fourteen studies reported on MCM, of which 10 reported on antivirals, 4 on vaccines and one on neutralising antibodies. Seven studies combined multiple study designs, 5 were laboratory studies and 2 were modelling studies. No interventional studies in humans were identified.

#### 3.4.6 Public Health and Social Measures

Only one study reporting on public health and social measures was identified. This was a cross‐sectional survey of knowledge, attitudes and practices among medical students in South America.

No studies were identified for vector control strategies, personal protective equipment and infection prevention control, or information, health education and behavioural interventions.

## 4 Discussion

This EGM highlighted that most of the published evidence identified on OROV was based on cross‐sectional and descriptive studies reporting on epidemiology and sero‐surveillance in humans, followed by laboratory studies on viral genomics. The majority of descriptive studies originated from South and Central America and the Caribbean, where the virus is endemic. Limited evidence was found on vector surveillance, viral pathogenesis, diagnostics and immune correlates of protection. No experimental studies on potential therapeutics or vaccines conducted in humans were identified.

Only 25 studies were identified for diagnostics. Since the virus tends to circulate in more remote areas with limited resources, accurate diagnostics (molecular and/or serological) may not be readily available, which may have led to OROV infection being misdiagnosed or overlooked. The recent 2024 outbreak in Brazil provided in vitro evidence of enhanced viral pathogenicity and the potential for re‐infection with a new oropouche viral reassortant [28], but otherwise the number of studies on host immune responses, viral pathogenesis and immune correlates of protection were found to be limited.

The lack of available evidence for MCM for OROV (particularly experimental and clinical studies in humans), may be due to the absence of adequate animal models for OROV infection and disease to allow pre‐clinical studies [29]. The absence of these studies also belies the fact that the virus was previously thought to cause a mild and self‐limiting infection and emphasises past lack of global scientific engagement with this virus.

### 4.1 Limitations

As this mapping review was conducted at pace, following rapid methodologies, we excluded any studies that did not directly study OROV to ensure a focused review question. Similarly, we did not include terms for vectors in the search strategy. As a result, entomological studies that assessed relevant vectors without reference to OROV were not included. Therefore, there may be findings from other studies that could be relevant to understanding OROV vectors, their ecology and vector control strategies that have not been included in the EGM.

The main methodological limitation of our study is that we followed streamlined methodologies, such as full‐text screening, coding and data extraction being done by one reviewer and checked by another rather than being done independently by two reviewers. Potential inconsistencies between reviewers were minimised through piloting and quality assurance processes. In addition, we mitigated the risk of missing potential relevant primary studies due to individual screening by consulting with experts from the Bunyaviricetes CORC and by screening the lists of relevant and reviews.

This mapping review exercise was conducted to complement a WHO/UKHSA CORC workshop of global experts aimed at developing an R&D roadmap for OROV [30]. The findings of the EGM were consistent with the key topics for research prioritisation identified in the roadmap. These include recommendations to:

1. Identify environmental drivers of vector breeding and urban spread
2. Study vector competence, reproduction and behaviour
3. Conduct genomic surveillance of circulating variants, and of genetic factors determining pathogenicity
4. Assess population exposure by seroprevalence studies
5. Identify serological markers that can distinguish between recent and historic infection
6. Develop rapid point of care diagnostics
7. Develop reference materials from endemic regions
8. Develop preclinical models, including challenge models
9. Advance therapeutic and vaccine candidates to preclinical testing, prioritising those with cross‐strain protection.

Simultaneous utilization of the EGM and R&D roadmap compiled by field experts has allowed the formulation and prioritisation of the most pressing research questions to counter the public health threat from OROV and help contain epidemics.

Independent of our study, an EGM for OROV was conducted by colleagues at PAHO, as part of a consultation meeting convened to develop a research agenda for the characterisation of OROV and its public health implications [31]. Whilst there were differences between the two EGMs, including differences in the type of studies included (the PAHO EGM was not limited to primary studies and included systematic reviews), how areas of research were categorised (the PAHO EGM centred around 5 different themes: vertical transmission, vectors and reservoirs, epidemiology and surveillance, laboratory, and clinical aspects) and how the studies were mapped (the PAHO EGM categorised the studies by population group rather than geographical area), the main findings were similar, in particular highlighting the need for additional research into clinical trials and MCM. The PAHO EGM also highlighted that studies among pregnant women and children were scarce. They concluded with slightly different emphasis on clinical based research, such as defining the case definitions to aid early diagnosis and management, and longitudinal studies to investigate its long‐term sequelae and the risk of vertical transmission.

The development and use of our EGM highlights the value of streamlined systemic methods for identifying gaps in evidence, particularly given the rapid expansion of OROV into new regions. Similar methods of combining systematic evidence mapping with expert‐identified clinical research priorities have been piloted for other outbreak scenarios, highlighting the utility of this complimentary approach as a tool for research prioritisation [32]. This EGM, used on its own or together with other prioritisation tools, will help decision‐makers visualise what evidence already exists, and identifies gaps, such as limited data on new vectors, changing ecology, or inconsistent surveillance systems. Together with contributions of global experts and the published R&D roadmap, the EGM will shape priority questions, guide investment decisions, inform decision‐making and strengthen preparedness for OROV as a priority pathogen for epidemic and pandemic responses.

## Author Contributions

**Sowsan F. Atabani:** conceptualisation, investigation, writing – original draft, writing – review and editing. **Matthew Bosworth:** methodology, investigation, visualisation. **Amy Bobbins:** investigation, visualisation, writing – original draft, writing – review and editing. **Beti Evans:** investigation, visualisation, editing. **Zalaya Simmons:** investigation. **Nicola Pearce‐Smith:** methodology, investigation, writing – review and editing. **Julia Tree:** writing – review and editing. **Charles Dearman:** conceptualisation, project administration, writing – review and editing. **Yper Hall:** conceptualisation, writing – review and editing. **Daphne Duval:** conceptualisation, methodology, supervision, writing – original draft, writing – review and editing.

## Funding

The authors have nothing to report.

## Conflicts of Interest

The authors declare no conflicts of interest.

## Supporting information


Supporting Information S1

Supporting Information S2

## Data Availability

The data that support the findings of this study are available in (repository name) at (DOI/URL), reference number (reference number). These data were derived from the following resources available in the public domain: (list resources and URLs).
