# Supplementary material for: Mapping the Evidence for Oropouche Virus to Inform Research Prioritisation
Source: Rev Med Virol. 2026 Oct 24;36(6):e70222. doi: 10.1002/rmv.70222 (PMC13695555; doi:10.1002/rmv.70222)
Supplement: Supplementary file 1 [file RMV-36-e70222-s002.docx]

**Supplementary material – Mapping the evidence for Oropouche Virus: an interactive evidence gap map to inform research prioritization**

Sowsan F Atabani, Matthew Bosworth, Amy Bobbins, Beti Evans, Zalaya Simmons, Nicola Pearce-Smith, Julia A Tree, Charles Dearman, Yper Hall, Isabel Oliver and Daphne Duval

**Supplementary material 1. Protocol**

The protocol was produced before the literature search began, specifying the review question and the inclusion and exclusion criteria.

The modifications made to the protocol were all related to the evidence gap map framework (see corresponding section).

**Review question:** What evidence is available on the Oropouche virus?

****Inclusion and exclusion criteria****

Review eligibility criteria are summarised in Table 1.

Table S1. Inclusion and exclusion criteria

|  | **Included** | **Excluded** |
| --- | --- | --- |
| Population | Human and animal studies |  |
| Concept | Any primary studies related to Oropouche virus, including (but not limited to):   - epidemiology and surveillance in humans - vector and ecology - virology and pathogenesis - diagnostics - medical countermeasures - public health and social measures |  |
| Context | No limitations on settings or countries |  |
| Language | No limitations |  |
| Date of publication | From database inception to 4 or 5 June 2025 |  |
| Study design | All primary study designs, including observational and studies in laboratory settings  Modelling studies | Guidelines  Opinion pieces  Systematic or narrative reviews |
| Publication type | Peer-reviewed articles  Preprints  Grey literature (such as reports published by public health agencies and non-governmental organisations, but excluding conference abstracts, theses and books) | Conference abstracts  Theses  Books and book chapters |

**Sources searched**

- Databases used for the literature search (initial searches conducted on 6 and 7 February 2025; search update conducted on 5 June 2025): Ovid Medline All (1946 to 4 June 2025), Ovid Embase (1974 to 4 June 2025), Ovid Global Health (1910 to 4 June 2025), Web of Science Core Collection, Science Citation Index (1970 to 5 June 2025), medRxiv (2019 to 5 June 2025), bioRxiv (2013 to 5 June 2025), LILACS Plus (1986 to 5 June 2025)
- Grey literature searching for non-indexed publications (initial searches conducted on 12 and 13 February 2025; search update conducted on 5 June 2025): [Pan American Health Organization (PAHO) Institutional Repository for Information Sharing (IRIS)](https://iris.paho.org/), [Caribbean Public Health Agency (CARPHA) evidence portal](https://carphaevidenceportal.bvsalud.org/), [Redalyc](https://redalyc.org/), and websites from the [WHO](https://www.who.int/), [Centers for Disease Control and Prevention](https://www.cdc.gov/) (CDC), [PAHO](https://www.paho.org/en) and [European Centre for Disease Prevention and Control](https://www.ecdc.europa.eu/en) (ECDC).

Additional sources of evidence included:

- Reference lists of relevant systematic reviews
- Consultation with experts

**Search strategies**

The initial database searches were conducted for papers published between database inception and 5 or 6 February 2025 (search dates: 6 and 7 February 2025, respectively) and updated on 5 June 2025. Search strategies were drafted by an information scientist and peer-reviewed by a second information scientist (see Supplementary material 2 for the search strategies used).

Grey literature searches were initially conducted by an information scientist on 12 and 13 February 2025 and updated on 5 June 2025, using the search phrase “Oropouche or OROV”.

On 14 July 2025, all included studies were checked for any retractions, and 8 preprints were examined to establish if they had subsequently been published as a peer reviewed paper.

**Screening**

Results from the database searches were downloaded into Endnote, then duplicates were removed using [Deduklick](https://www.risklick.ch/deduklick) (an automated AI deduplication tool). Results from the grey literature searches were deduplicated using Endnote. Final results were imported into EPPI-Reviewer web version, where further deduplication was conducted before screening.

Title and abstract screening of records identified was completed in duplicate by 2 reviewers for 20% of the studies, with disagreements resolved by discussion. The remainder were screened by one reviewer in EPPI-Reviewer web version. The priority screening system was used to screen the original database search results, which uses machine learning to prioritise the order of references by their likelihood of inclusion based on prior screening decisions (all studies were screened by a human reviewer).

In an initial pilot phase, 5% of the studies included on title and abstract were screened on full text in duplicate by 2 reviewers. The remainder were screened on full text by one reviewer and checked by a second using EPPI-Reviewer web version. Disagreements were resolved by discussion, with a third reviewer involved if consensus could not be reached.

To enable screening of non-English language papers, Google translate was used to translate papers into English.

Screening of the reference lists of relevant systematic reviews was done by one reviewer.

**Data extraction**

As this was a rapid EGM, only information required for coding the studies (area of research, study design, and geographic region) was extracted directly into EPPI-Reviewer.

In an initial pilot phase, 10% of the included studies were coded in duplicate by 2 reviewers with disagreements resolved by discussion. The remainder was conducted by one reviewer and checked by a second.

To enable data extraction from non-English language papers, Google Translate was used to translate papers into English.

**Critical appraisal**

For this rapid EGM, it was agreed when drafting the protocol that critical appraisal would not be performed as the main aim of this work was to identify and categorise the evidence identified^[1]^.

**EGM framework and visual synthesis**

Visual synthesis was performed by generating an interactive EGM using EPPI-Mapper, using the coding extracted to represent the evidence identified on Oropouche virus by area of research and by study design, with a third dimension to represent the geographic region where the study was conducted.

Area of research (2 levels of coding: parent and child codes):

- epidemiology and surveillance in humans:
- natural history and clinical manifestations
- modes of transmission
- surveillance in humans
- other
- vector and ecology:
- vector competence
- surveillance in arthropod vectors
- surveillance in animal reservoirs
- climate and environmental factors
- virology and pathogenesis:
- host immune responses
- viral genomics
- virus tropism
- viral replicative mechanisms
- other
- diagnostics:
- serological diagnostics
- molecular diagnostics
- clinical diagnostics
- viral sequencing
- assay standards
- other
- medical countermeasures:
- antivirals
- neutralising antibodies
- vaccines
- public health and social measures
- vector control strategies
- personal protective equipment and infection prevention control
- information, health education and behavioural interventions
- other

Study design (2 levels of coding: parent and child codes):

- experimental studies in humans:
- randomised controlled trials
- non-randomised controlled trials
- other trials
- observational studies in humans:
- longitudinal studies
- cross-sectional studies
- other analytical studies
- outbreak investigations
- case reports and case series
- other descriptive studies
- laboratory studies:
- animal studies
- entomological studies
- in vitro studies
- genome sequencing studies
- other laboratory studies
- zoonotic field studies
- modelling studies
- studies with multiple designs (for example, a paper that reported on an observational study in humans and a laboratory study or zoonotic field study)

Geographic region (1 level of coding):

- Africa
- Asia and Oceania
- Central America and the Caribbean
- Europe
- North America
- South America

**Modifications made to the protocol**

- The original subcategory ‘cohort studies’ was changed to ‘longitudinal studies’ because no cohort studies were identified. However, some studies were identified that collected repeated samples from a subgroup of study participants or from a particular area over a period of time, which were categorised as longitudinal studies.
- The subcategory ‘other’ was added for some areas of research to categorise studies that did not fit into the existing subcategories.
- The subcategory ‘viral replicative mechanisms’ was added under virology and pathogenesis. This is because 9 studies had been identified on viral replicative mechanisms and, following discussion among the review team and with topic experts, it was agreed to add this additional subcategory in order to group these studies together in a more meaningful way.
- The subcategory ‘natural history’ was renamed to ‘natural history and clinical manifestations’ to provide clarity about the type of studies included in this subcategory.
- The subcategory ‘clinical diagnostics’ was also added to highlight the lack of research in this area.

**Supplementary material 2. Search strategies**

Search strategy for Ovid Medline ALL (1946 to 4 June 2025)

1. oropouche*.tw,kf.

2. sloth fever*.tw,kf.

3. OROV.tw,kf.

4. Bunyaviridae Infections/

5. 1 or 2 or 3 or 4

Search strategy for Ovid Embase (1974 to 4 June 2025)

1. oropouche*.tw,kf.

2. sloth fever*.tw,kf.

3. OROV.tw,kf.

4. oropouche orthobunyavirus/ or oropouche virus/

5. bunyavirus infection/

6. 1 or 2 or 3 or 4 or 5

Search strategy for Ovid Global Health (1910 to 4 June 2025)

1. oropouche.af.

2. sloth fever.af.

3. OROV.af.

4. 1 or 2 or 3

Search strategy for Web of Science Citation Index (1970 to 5 June 2025)

1. TS=(oropouche*) or TS=("sloth fever*") or TS=(OROV)

Search strategy for medRxiv (June 2019 to 5 June 2025)

1. oropouche OR OROV

Search strategy for bioRxiv (2013 to 5 June 2025)

1. oropouche OR OROV

Search strategy for LILACS Plus (1986 to 5 June 2025)

1. oropouche or OROV or mh:("Bunyaviridae Infections")

**Supplementary material 3. List of included studies (n = 269)**

1. Acrani GO, Gomes R, Proenca-Modena Jose L, et al. Apoptosis induced by Oropouche virus infection in HeLa cells is dependent on virus protein expression. Virus research **2010**; 149:56–63.

2. Acrani GO, Tilston-Lunel Natasha L, Spiegel M, et al. Establishment of a minigenome system for Oropouche virus reveals the S genome segment to be significantly longer than reported previously. The Journal of general virology **2015**; 96:513–23.

3. Adhikari UK, Tayebi M, Rahman MM. Immunoinformatics approach for epitope-based peptide vaccine design and active site prediction against polyprotein of emerging Oropouche virus. Journal of immunology research **2018**; 2018:6718083.

4. Aguilar PV, Barrett Alan D, Saeed Mohammad F, et al. Iquitos virus: a novel reassortant Orthobunyavirus associated with human illness in Peru. PLoS neglected tropical diseases **2011**; 5:e1315.

5. Almeida GM, Souza Juliano P, Mendes Niele D, et al. Neural infection by Oropouche virus in adult human brain slices induces an inflammatory and toxic response. Frontiers in neuroscience **2021**; 15:674576.

6. Alva-Urcia C, Aguilar-Luis Miguel A, Palomares-Reyes C, et al. Emerging and reemerging arboviruses: A new threat in Eastern Peru. PloS one **2017**; 12:e0187897.

7. Alvarez-Falconi P, Pedro P, Ríos R, Bessy A. Oropuche fever outbreak in Bagazan,San Martín, Peru: epidemiological evaluation, gastrointestinal and hemorrhagic manifestations. Revista de Gastroenterología del Perú **2010**; 30:334–40.

8. Alves Araujo FA, Lima PC, Andrade MA, Jayme VdS, Ramos DG, Silveira SLd. Seroprevalence of antibodies to arboviruses of public health importance in wild birds, Brazil - 2007 and 2008. Ciência Animal Brasileira **2012**; 13:115–23.

9. Alves da Silva Queiroz J, Botelho-Souza Luan F, Nogueira-Lima Felipe S, et al. Phylogenetic characterization of arboviruses in patients suffering from acute fever in Rondonia, Brazil. Viruses **2020**; 12.

10. Alves do Nascimento V, Santos Joao Hugo A, Monteiro Dana Cristina da S, et al. Oropouche virus detection in saliva and urine. Memorias do Instituto Oswaldo Cruz **2020**; 115:e190338.

11. Anderson CR, Spence L, Downs WG, Aitken TH. Oropouche virus: a new human disease agent from Trinidad, West Indies. The American journal of tropical medicine and hygiene **1961**; 10:574–8.

12. Andreolla AP, Borges Alessandra A, Nagashima S, et al. Development of monoclonal antibodies against oropouche virus and its applicability to immunohistochemical diagnosis. Virology journal **2024**; 21:81.

13. Aquino VH, Figueiredo Luiz Tadeu M. Linear amplification followed by single primer polymerase chain reaction to amplify unknown DNA fragments: complete nucleotide sequence of Oropouche virus M RNA segment. Journal of virological methods **2004**; 115:51–7.

14. Aquino VH, Moreli ML, Moraes F, L T. Analysis of oropouche virus L protein amino acid sequence showed the presence of an additional conserved region that could harbour an important role for the polymerase activity. Archives of virology **2003**; 148:19–28.

15. Araujo R, Dias LB, Araujo MT, Pinheiro F, Oliva OF. Ultrastructural changes in the hamster liver after experimental inoculation with Oropouche arbovirus (type BeAn 19991). Revista do Instituto de Medicina Tropical de São Paulo **1978**; 20:45–54.

16. Baer K, Arora I, Kimbro J, et al. Iquitos virus in traveler returning to the United States from Ecuador. Emerging infectious diseases **2024**; 30:2447–51.

17. Baisley KJ, Watts DM, Munstermann LE, Wilson ML. Epidemiology of endemic Oropouche virus transmission in upper Amazonian Peru. The American Journal of Tropical Medicine and Hygiene **1998**; 59:710–6.

18. Bandeira AC, Pereira Felicidade M, Leal A, et al. Fatal Oropouche virus infections in nonendemic region, Brazil, 2024. Emerging infectious diseases **2024**; 30:2370–4.

19. Barbiero A, Formica G, Mantovani Rebeca P, et al. Persistent Oropouche virus viremia in two travellers returned to Italy from Cuba, July 2024. Journal of travel medicine **2024**; 32:taae148.

20. Barbosa NS, Concha Juan O, daSilva Luis LP, Crump Colin M, Graham Stephen C. Oropouche virus glycoprotein topology and cellular requirements for glycoprotein secretion. Journal of virology **2023**; 97:e0133122.

21. Barbosa NS, Mendonca Leila R, Dias Marcos VS, et al. ESCRT machinery components are required for Orthobunyavirus particle production in Golgi compartments. PLoS pathogens **2018**; 14:e1007047.

22. Barreto de Almeida MA, Santos Edmilson d, Cardoso Jáder da C, et al. Detection of antibodies against Icoaraci, Ilhéus, and Saint Louis Encephalitis arboviruses during yellow fever monitoring surveillance in non-human primates (Alouatta caraya) in southern Brazil. Journal of Medical Primatology **2019**; 48:211–7.

23. Batista PM, Andreotti R, Almeida Paulo Silva d, et al. Detection of arboviruses of public health interest in free-living New World primates (Sapajus spp.; Alouatta caraya) captured in Mato Grosso do Sul, Brazil. Revista da Sociedade Brasileira de Medicina Tropical **2013**; 46:684–90.

24. Batista PM, Andreotti R, Chiang Jannifer O, Ferreira Milene S, Vasconcelos Pedro Fernando da C. Seroepidemiological monitoring in sentinel animals and vectors as part of arbovirus surveillance in the state of Mato Grosso do Sul, Brazil. Revista da Sociedade Brasileira de Medicina Tropical **2012**; 45:168–73.

25. Batista Salgado B, de Jesus Maues Fabio C, Pereira Renato L, et al. Prevalence of arbovirus antibodies in young healthy adult population in Brazil. Parasites & vectors **2021**; 14:403.

26. Bello-Rodriguez Berta M, Vega-Jimenez J, Canete R, Rodriguez-Morales Alfonso J. Emergence of Oropouche virus infection in Matanzas, Cuba, 2024. The Journal of infection **2025**; 90:106470.

27. Benitez AJ, Alvarez M, Perez L, et al. Oropouche Fever, Cuba, May 2024. Emerging infectious diseases **2024**; 30:2155–9.

28. Bernardes Terzian AC, Bronzoni Roberta Vieira de M, Drumond Betânia P, et al. Sporadic Oropouche infection, Acre, Brazil. Emerging Infectious Diseases **2009**; 15:348.

29. Bernardes Terzian AC, Mondini A, Bronzoni Roberta Vieira de M, et al. Detection of Saint Louis encephalitis virus in dengue-suspected cases during a dengue 3 outbreak. Vector borne and zoonotic diseases **2011**; 11:291–300.

30. Bernardes-Terzian AC, de-Moraes-Bronzoni Roberta V, Drumond Betania P, et al. Sporadic oropouche virus infection, acre, Brazil. Emerging infectious diseases **2009**; 15:348–50.

31. Borborema CA, Pinheiro FP, Albuquerque BC, da Rosa AP, da Rosa JF, Dourado HV. 1st occurrence of outbreaks caused by Oropouche virus in the State of Amazonas. Revista do Instituto de Medicina Tropical de São Paulo **1982**; 24:132–9.

32. Bueno da Silva Menegatto M, Ferraz Ariane C, Lima Rafaela Lameira S, et al. Oropouche virus infection induces ROS production and oxidative stress in liver and spleen of mice. The Journal of general virology **2023**; 104.

33. Caceda ER, Kochel Tadeusz J. Application of modified shell vial culture procedure for arbovirus detection. PloS one **2007**; 2:e1034.

34. Cardoso BF, Serra Otacilia P, Heinen Leticia Borges da S, et al. Detection of Oropouche virus segment S in patients and in Culex quinquefasciatus in the state of Mato Grosso, Brazil. Memorias do Instituto Oswaldo Cruz **2015**; 110:745–54.

35. Carvalho ARV, Reis Jose Diogo E, Gomes Paulo Wender P, et al. Untargeted-based metabolomics analysis and in vitro/in silico antiviral activity of extracts from Phyllanthus brasiliensis (Aubl.) Poir. Phytochemical analysis : PCA **2023**; 34:869–83.

36. Carvalho de Oliveira Silva J, Siqueira Igor Rodrigo F, Dornelas Leormando F, et al. Occurrence of arbovirus infections in two riverine populations in the municipality of Humaita, Amazonas, Brazil. Revista da Sociedade Brasileira de Medicina Tropical **2024**; 57:e004032024.

37. Carvalho VL, Azevedo Raimunda SS, Carvalho Valeria L, et al. Arbovirus outbreak in a rural region of the Brazilian Amazon. Journal of Clinical Virology **2022**; 150-151:105155.

38. Casseb AR, Silva Sandro P, Casseb Livia Medeiros N, Chiang Jannifer O, Martins Lívia C, Vasconcelos Pedro Fernando C. Prevalence of Arbovirus antibodies against the family Bunyaviridae in water buffaloes. Ciência Animal Brasileira **2015**; 16:428–36.

39. Castilletti C, Huits R, Mantovani Rebeca P, Accordini S, Alladio F, Gobbi F. Replication-competent Oropouche virus in semen of traveler returning to Italy from Cuba, 2024. Emerging infectious diseases **2024**; 30:2684–6.

40. Castilletti C, Mori A, Matucci A, et al. Oropouche fever cases diagnosed in Italy in two epidemiologically non-related travellers from Cuba, late May to early June 2024. Eurosurveillance **2024**; 29.

41. Castro S, Banda L, Cabellos D, et al. Oropuche fever outbreak in two villages of the region Cajamarca, Peru, 2011. Revista Peruana de Epidemiología **2013**; 17:1–6.

42. Catenacci LS, Ferreira M, Martins LC, et al. Surveillance of Arboviruses in primates and sloths in the Atlantic Forest, Bahia, Brazil. EcoHealth **2018**; 15:777–91.

43. Catenacci LS, Ferreira Milene S, Fernandes D, et al. Individual, household and environmental factors associated with arboviruses in rural human populations, Brazil. Zoonoses and Public Health **2021**; 68:203–12.

44. Chiang JO, Azevedo Rafael S, Justino Maria CA, et al. Neurological disease caused by Oropouche virus in northern Brazil: should it be included in the scope of clinical neurological diseases? Journal of Neurovirology **2021**; 27:626–30.

45. Ciuoderis KA, Berg Michael G, Perez Lester J, et al. Oropouche virus as an emerging cause of acute febrile illness in Colombia. Emerging microbes & infections **2022**; 11:2645–57.

46. Coimbra LD, Shimizu Jacqueline F, Nagai A, et al. Lysergol exerts potent antiviral activity against the emerging Oropouche virus in vitro. Antiviral research **2025**; 238:106171.

47. Cola JP, Brioschi Dos S, Ana P, et al. A case report of potential intrapartum transmission of Oropouche virus in an outbreak in Brazil. The Pediatric infectious disease journal **2025**.

48. Cola JP, Dos Santos Ana Paula B, Zanotti Raphael L, et al. Maternal and fetal implications of Oropouche fever, Espirito Santo state, Brazil, 2024. Emerging infectious diseases **2025**; 31:645–51.

49. Colavita F, Carletti F, D'Abramo A, Nicastri E, Maggi F. Prolonged detection of Oropouche virus RNA in whole blood samples. The Lancet Infectious Diseases **2025**; 25:e11–e2.

50. Concha JO, Gutierrez K, Barbosa N, et al. Rab27a GTPase and its effector Myosin Va are host factors required for efficient Oropouche virus cell egress. PLoS pathogens **2024**; 20:e1012504.

51. Cruz CD, Espejo V, Espinoza A, et al. Co-circulation of 2 Oropouche virus lineages during outbreak, Amazon region of Peru, 2023-2024. Emerging infectious diseases **2025**; 31:879–81.

52. Curtinhas de Lima R, Dias Helver G, de Souza Thiara Manuele A, et al. Oropouche virus exposure in febrile patients during Chikungunya virus introduction in the state of Amapa, Amazon region, Brazil. Pathogens **2024**; 13.

53. da Costa Vasconcelos PF, Rosa Jorge Fernando Soares Travassos d, Guerreiro Sueli C, Dégallier N, Rosa Elizabeth Salbé Travassos d, Rosa Amélia Paes de Andrade Travassos d. 1st register of an epidemic caused by oropouche virus in the states of Maranhão and Goiás, Brazil. Revista do Instituto de Medicina Tropical de Sao Paulo **1989**; 31:271–8.

54. da Silva Ferreira R, de Toni Aquino da Cruz LC, de Souza Vilma J, et al. Insect-specific viruses and arboviruses in adult male culicids from Midwestern Brazil. Infection, genetics and evolution **2020**; 85:104561.

55. das Neves Martins FE, Chiang Jannifer O, Nunes Bruno Tardelli D, et al. Newborns with microcephaly in Brazil and potential vertical transmission of Oropouche virus: a case series. The Lancet Infectious Diseases **2025**; 25:155–65.

56. Daudt-Lemos M, Ramos-Silva A, Faustino R, et al. Rising incidence and spatiotemporal dynamics of emerging and reemerging Arboviruses in Brazil. Viruses **2025**; 17.

57. de Almeida Neves Azevedo E, Silva Alexandre Fd, Silva Veronica Gd, et al. Genomic and phenotypic characterization of the Oropouche virus strain implicated in the 2022-24 large-scale outbreak in Brazil. Journal of medical virology **2024**; 96:e70012.

58. de Armas Fernández JR, García CEP, Herrera BA, et al. Incidence of Guillain-Barre syndrome in Cuba before and during the Oropouche virus emergency, 2018-2024. JOURNAL OF NEUROLOGY **2025**; 272.

59. de Armas Fernández JR, Pena Garcia Carilda E, Acosta H, et al. Report of an unusual association of Oropouche Fever with Guillain-Barre syndrome in Cuba, 2024. European Journal of Clinical Microbiology & Infectious Diseases **2024**; 43:2233–7.

60. de Castro FL, Brustolini Otavio Jose B, Geddes Victor Emmanuel V, et al. Modulation of HERV expression by four different encephalitic arboviruses during infection of human primary astrocytes. Viruses **2022**; 14.

61. de Freitas Rodrigues Ribeiro B, Barreto Andre Rodrigues F, Pessoa A, et al. Congenital oropouche in humans: clinical characterization of a possible new teratogenic syndrome. Viruses **2025**; 17.

62. de Lima STS, Hua X, Claro Ingra M, et al. Molecular epidemiology of Oropouche virus, Ceara State, Brazil, 2024. Emerging infectious diseases **2025**; 31:838–42.

63. de Melo Iani FC, Pereira Felicidade M, de Oliveira Elaine C, et al. Travel-associated international spread of Oropouche virus beyond the Amazon. Journal of travel medicine **2025**; 32.

64. de Mendonca SF, Baldon Livia VR, Todjro Yaovi MH, et al. Oropouche orthobunyavirus in Urban Mosquitoes: Vector Competence, Coinfection, and Immune System Activation in Aedes aegypti. Viruses **2025**; 17.

65. de Mendonca SF, Rocha Marcele N, Ferreira Flavia V, et al. Evaluation of Aedes aegypti, Aedes albopictus, and Culex quinquefasciatus mosquitoes competence to Oropouche virus Infection. Viruses **2021**; 13.

66. de Oliveira E, Azevedo Raimunda do Socorro S, Coelho-Dos-Reis Jordana G, et al. IFN-alpha as a time-sensitive biomarker during Oropouche virus infection in early and late seroconverters. Scientific Reports **2019**; 9:17924.

67. de Paula Pinheiro F, Bensabath G, Andrade Amelia Homobono Paes d, et al. Infectious diseases along Brazil's trans-amazon highway: surveillance and research. Bulletin of the Pan American Health Organization **1974**; 8:111–22.

68. de Souza Bastos M, Figueiredo Luiz Tadeu M, Naveca Felipe G, et al. Identification of Oropouche Orthobunyavirus in the cerebrospinal fluid of three patients in the Amazonas, Brazil. The American journal of tropical medicine and hygiene **2012**; 86:732–5.

69. de Souza Bastos M, Lessa N, Naveca Felipe G, et al. Detection of Herpesvirus, Enterovirus, and Arbovirus infection in patients with suspected central nervous system viral infection in the Western Brazilian Amazon. Journal of medical virology **2014**; 86:1522–7.

70. de Souza Costa MC, Siqueira M, L M, et al. Arbovirus investigation in patients from Mato Grosso during Zika and Chikungunya virus introdution in Brazil, 2015-2016. Acta tropica **2019**; 190:395–402.

71. de Souza Luna LK, Rodrigues Alcir H, Santos Rodrigo Ivo M, et al. Oropouche virus is detected in peripheral blood leukocytes from patients. Journal of medical virology **2017**; 89:1108–11.

72. Degallier N, Travassos da Rosa APA, Vasconcelos PFC. Modifications of arbovirus transmission in relation to construction of dams in the Brazilian Amazon. Ciencia e Cultura **1992**; 44:124–35.

73. Deiana M, Malago S, Mori A, et al. Full genome characterization of the first Oropouche virus isolate imported in Europe from Cuba. Viruses **2024**; 16.

74. del Valle-Mendoza J, Vasquez-Achaya F, Aguilar-Luis Miguel A, et al. Unidentified dengue serotypes in DENV positive samples and detection of other pathogens responsible for an acute febrile illness outbreak 2016 in Cajamarca, Peru. BMC research notes **2020**; 13:467.

75. Delatorre E, de Mendonca Gabriela C, Gatti Felipe D, et al. Emergence of Oropouche Virus in Espirito Santo State, Brazil, 2024. Emerging infectious diseases **2025**; 31:1178–88.

76. Dev Sharma P, Alhudhaibi Abdulrahman M, Al Noman A, Abdallah Emad M, Taha Tarek H, Sharma H. Systems biology-driven discovery of host-targeted therapeutics for Oropouche virus: integrating network pharmacology, molecular docking, and drug repurposing. Pharmaceuticals **2025**; 18.

77. Dias HG, de Lima Raquel C, Barbosa Luciana S, et al. Retrospective molecular investigation of Mayaro and Oropouche viruses at the human-animal interface in West-central Brazil, 2016-2018. PloS one **2022**; 17:e0277612.

78. Dias HG, Familiar-Macedo D, Garrido Ingrid O, Dos S, Flavia B, Pauvolid-Correa A. Exposure of domestic animals to Mayaro and Oropouche viruses in urban and peri-urban areas of West-Central Brazil. One health outlook **2024**; 6.

79. Dixon KE, Llewellyn CH, Travassos da Rosa APA, Travassos da Rosa JF. A multidisciplinary program of infectious disease surveillance along the Transamazon Highway in Brazil: Epidemiology of arbovirus infections. Bulletin of the Pan American Health Organization **1981**; 15:11 EP–25.

80. Dixon KE, Travassos da Rosa AP, Travassos da Rosa JF, Llewellyn CH. Oropouche virus. II. Epidemiological observations during an epidemic in Santarem, Para, Brazil in 1975. The American journal of tropical medicine and hygiene **1981**; 30:161–4.

81. do Carmo Alves Martins V, Bastos Michele de S, Ramasawmy R, et al. Clinical and virological descriptive study in the 2011 outbreak of dengue in the Amazonas, Brazil. PloS one **2014**; 9:e100535.

82. do Carmo Silva L, Silva Diego Michel Fernandes D, Calassa Igor Marques C, et al. Fast and visual RT-LAMP assay for detection of oropouche virus. European journal of clinical microbiology & infectious diseases **2025**.

83. do Socorro da Silva Azevedo R, Nunes Marcio Roberto T, Chiang Jannifer O, et al. Reemergence of Oropouche fever, northern Brazil. Emerging infectious diseases **2007**; 13:912–5.

84. dos Santos Fonseca LM, Carvalho Rejane H, Bandeira Antonio C, Sardi Silvia I, Campos Gubio S. Oropouche virus detection in febrile patients' saliva and urine samples in Salvador, Bahia, Brazil. Japanese journal of infectious diseases **2020**; 73:164–5.

85. dos Santos Peinado R, Saivish Marielena V, Menezes Gabriela de L, et al. The search for an antiviral lead molecule to combat the neglected emerging Oropouche virus. Current research in microbial sciences **2024**; 6:100238.

86. Durango-Chavez HV, Toro-Huamanchumo Carlos J, Silva-Caso W, et al. Oropouche virus infection in patients with acute febrile syndrome: Is a predictive model based solely on signs and symptoms useful? PloS one **2022**; 17:e0270294.

87. Elbadry Maha A, Duraes-Carvalho R, Blohm Gabriela M, et al. Orthobunyaviruses in the Caribbean: Melao and Oropouche virus infections in school children in Haiti in 2014. PLoS neglected tropical diseases **2021**; 15:e0009494.

88. Estudio interinstitucional desarrollado por las instituciones del Ministerio. Etiologic profile of febrile syndrome in areas of high risk of disease transmission infectious diseases of impact on public health in Peru, 2000-2001. Revista Peruana de Medicina Experimental y Salud Pública **2005**; 22:165–74.

89. Ferdous Z, Dieme C, Sproch H, et al. Multiple bloodmeals enhance dissemination of arboviruses in three medically relevant mosquito genera. Parasites & vectors **2024**; 17:432.

90. Figueiredo Magalhães de Brito MT, Tinara Leila de Souza A, da Silva Pinto D. Seroepidemiology of arbovirus in communities living under the influence of the lake of a hydroelectric dam in Brazil. Cadernos Saúde Coletiva **2018**; 26:1–6.

91. Fischer C, Fruhauf A, Inchauste L, et al. The spatiotemporal ecology of Oropouche virus across Latin America: a multidisciplinary, laboratory-based, modelling study. The Lancet Infectious diseases **2025**.

92. Forato J, Meira CA, Claro IM, et al. Molecular epidemiology of Mayaro virus among febrile patients, Roraima State, Brazil, 2018-2021. Emerging Infectious Diseases **2024**; 30:1013–6.

93. Forshey BM, Guevara C, Laguna-Torres VA, et al. Arboviral etiologies of acute febrile illnesses in Western South America, 2000-2007. PLoS neglected tropical diseases **2010**; 4:e787.

94. Freitas RB, Pinheiro FP, Santos MAV, Travassos Da Rosa APA, Travassos Da Rosa JFS, Freitas EN. Epidemic of Oropouche virus disease in eastern Para State, Brazil, in 1979. Revista da FSESP **1980**; 25:59–72.

95. Gaillet M, Pichard C, Restrepo J, et al. Outbreak of Oropouche virus in French Guiana. Emerging infectious diseases **2021**; 27:2711–4.

96. Garcia F, Lima N, A S, et al. A case of vertical transmission of Oropouche virus in Brazil. New England Journal of Medicine **2024**; 391:2055–7.

97. García MP, Merino Nancy S, Figueroa D, et al. Detection of Oropouche viral circulation in Madre de Dios region, Peru (december 2015 to january 2016). Revista Peruana de Medicina Experimental y Salud Pública **2016**; 33:380–1.

98. Gibrail Marize M, Fiaccadori Fabiola S, Souza M, et al. Detection of antibodies to Oropouche virus in non-human primates in Goiania City, Goias. Revista da Sociedade Brasileira de Medicina Tropical **2016**; 49:357–60.

99. Gil-Mora J, Acevedo-Gutierrez LY, Betancourt-Ruiz Paola L, et al. Arbovirus antibody seroprevalence in the human population from Cauca, Colombia. The American journal of tropical medicine and hygiene **2022**; 107:1218–25.

100. Gomes da Costa V, de Rezende Feres Valeria C, Saivish Marielena V, de Lima Gimaque Joao B, Moreli Marcos L. Silent emergence of Mayaro and Oropouche viruses in humans in Central Brazil. International Journal of Infectious Diseases **2017**; 62:84–5.

101. Gomez-Camargo Doris E, Egurrola-Pedraza Jorge A, Cruz Cristopher D, et al. Evidence of Oropouche Orthobunyavirus infection, Colombia, 2017. Emerging infectious diseases **2021**; 27:1756–8.

102. Goncalves M, Luiz H, Vieira da Rocha Neto C, et al. Prevalence of arboviruses and other infectious causes of skin rash in patients treated at a tertiary health unit in the Brazilian Amazon. PLoS neglected tropical diseases **2022**; 16:e0010727.

103. Gourjault C, Pezzi L, Doudier B, et al. Persistence of Oropouche virus in body fluids among imported cases in France, 2024. The Lancet Infectious Diseases **2025**; 25:e64–e5.

104. Graf T, Delatorre E, do Nascimento F, et al. Expansion of Oropouche virus in non-endemic Brazilian regions: analysis of genomic characterisation and ecological drivers. The Lancet Infectious Diseases **2025**; 25:379 EP–89.

105. Gregorio Co AC, de Mendonca Gabriela C, Gatti Felipe D, et al. Unravelling the pathogenesis of Oropouche virus. The Lancet Infectious diseases **2025**; 25:E381–E2.

106. Grisales-Nieto JC, Luz Sergio Luiz B, Nascimento Valdinete Alves d, et al. First case of Oropouche fever detected in the international border region of the Colombian Amazon: clinical characteristics and molecular diagnosis. Memorias do Instituto Oswaldo Cruz **2024**; 119:e230221.

107. Guagliardo SJ, Martin S, Gould Carolyn V, et al. Estimation of incubation period for Oropouche virus disease among travel-associated cases, 2024-2025. Emerging infectious diseases **2025**; 31:1337–43.

108. Guevara C, Ampuero Julia S, Schilling M. Arthropod-borne and rodent-borne infections in Peru from 1990 to 2022. The Journal of infectious diseases **2025**; 231:S72–S9.

109. Gunter K, Omoga D, Bowen James M, et al. A reporter Oropouche virus expressing ZsGreen from the M segment enables pathogenesis studies in mice. Journal of virology **2024**; 98:e0089324.

110. Gutierrez B, Wise Emma L, Pullan Steven T, et al. Evolutionary dynamics of Oropouche virus in South America. Journal of virology **2020**; 94.

111. Hoch AL, Pinheiro FP, Roberts DR, Gomes ML. Laboratory transmission of Oropouche virus by Culex Quinquefasciatus Say. Bulletin of the Pan American Health Organization **1987**; 21:55–61.

112. Igloi Z, Soochit W, Munnink Bas BO, et al. Oropouche virus genome in semen and other body fluids from traveler. Emerging infectious diseases **2025**; 31:205–6.

113. Izurieta RO, Macaluso M, Watts Douglas M, et al. Anamnestic immune response to dengue and decreased severity of yellow fever. Journal of global infectious diseases **2009**; 1:111–6.

114. Jonkers AH, Spence L, Downs WG, Aitken THG, Tikasingh ES. Arbovirus studies in Bush Bush Forest, Trinidad, W.I., September 1959-December 1964. V. Virus isolations. American Journal of Tropical Medicine and Hygiene **1968**; 17:276–84.

115. Ladner JT, Savji N, Lofts L, et al. Genomic and phylogenetic characterization of viruses included in the Manzanilla and Oropouche species complexes of the genus Orthobunyavirus, family Bunyaviridae. The Journal of general virology **2014**; 95:1055–66.

116. Laroque PO, Valença-Montenegro Mônica M, Ferreira Débora RA, et al. Epidemiologic survey for arbovirus in galician capuchin monkeys (Cebus flavius) free living in Paraíba and captive capuchin monkey (Cebus libidinosus) from northeast Brazil. Pesquisa Veterinária Brasileira **2014**; 34:462–8.

117. Lavezzo LC, Santana VdS, Terzian ACB, et al. Arboviruses in blood donors: a study in the Amazon region and in a small city with a dengue outbreak. Transfusion Medicine **2010**; 20:278–9.

118. Leal CS, Carvalho Carlos Alberto M. In silico physicochemical characterization of fusion proteins from emerging Amazonian Arboviruses. Life **2023**; 13.

119. LeDuc JW, Hoch AL, Pinheiro FP, da Rosa AP. Epidemic Oropouche virus disease in northern Brazil. Bulletin of the Pan American Health Organization **1981**; 15:97–103.

120. Livonesi MC, De Sousa Ricardo LM, Badra Soraya J, Figueiredo Luiz TM. In vitro and in vivo studies of ribavirin action on Brazilian Orthobunyavirus. The American journal of tropical medicine and hygiene **2006**; 75:1011–6.

121. Livonesi MC, de Sousa Ricardo Luiz M, Badra Soraya J, Figueiredo Luiz Tadeu M. In vitro and in vivo studies of the Interferon-alpha action on distinct Orthobunyavirus. Antiviral research **2007**; 75:121–8.

122. Livonesi MC, Moro de Sousa RL, Moraes F, Luiz T. In vitro study of antiviral activity of mycophenolic acid on Brazilian orthobunyaviruses. Intervirology **2007**; 50:204–8.

123. Lopes-Ribeiro A, Araujo Franklin P, Oliveira Patricia de M, et al. In silico and in vitro arboviral MHC class I-restricted-epitope signatures reveal immunodominance and poor overlapping patterns. Frontiers in immunology **2022**; 13:1035515.

124. Lorenz C, Azevedo Thiago S, Virginio F, Aguiar Breno S, Chiaravalloti-Neto F, Suesdek L. Impact of environmental factors on neglected emerging arboviral diseases. PLoS neglected tropical diseases **2017**; 11:e0005959.

125. Maciel Feitoza LH, de Carvalho Luis Paulo C, da Silva Lucas R, et al. Influence of meteorological and seasonal parameters on the activity of Culicoides paraensis (Diptera: Ceratopogonidae), an annoying anthropophilic biting midge and putative vector of Oropouche Virus in Rondonia, Brazilian Amazon. Acta tropica **2023**; 243:106928.

126. Maciel Feitoza LH, Gasparelo Nadson Willian F, Meireles Anne Caroline A, et al. Integrated surveillance for Oropouche Virus: Molecular evidence of potential urban vectors during an outbreak in the Brazilian Amazon. Acta tropica **2025**; 261:107487.

127. Maier MA, Neurohr E-M, Barreto-Miranda I, Gabriel M, Gunther S, Belard S. Imported Oropouche fever to Germany in a returning traveller from Cuba. Infection **2024**; 53:753–4.

128. Mancon A, Gagliardi G, Giacomelli A, et al. Oropouche fever diagnosed in Milan, Italy in returning travellers from Rio de Janeiro, March 2024, and Cuba, July 2024. Journal of Travel Medicine **2024**; 31.

129. Mandova T, Saivish Marielena V, Menezes Gabriela de L, et al. Antiviral Activity and Molecular Dynamics Simulation of Hops Compounds against Oropouche Virus (Peribunyaviridae). Pharmaceutics **2023**; 15:2769.

130. Manock SR, Jacobsen Kathryn H, de Bravo Narcisa B, et al. Etiology of acute undifferentiated febrile illness in the Amazon basin of Ecuador. The American journal of tropical medicine and hygiene **2009**; 81:146–51.

131. Martins-Filho PR, Carvalho Thialla A, Dos S, Cliomar A. Spatiotemporal epidemiology of Oropouche fever, Brazil, 2015-2024. Emerging infectious diseases **2024**; 30:2196–8.

132. Martins-Filho PR, Carvalho Thialla A, Quintans-Junior Lucindo J, Araujo Adriano Antunes de S, Goes Marco Aurelio de O, Santos Cliomar Alves D. First Oropouche fever cases in a Northeastern Brazilian state, April to September 2024. Revista do Instituto de Medicina Tropical de Sao Paulo **2025**; 67:e27.

133. Martins-Luna J, Del Valle-Mendoza J, Silva-Caso W, et al. Oropouche infection a neglected arbovirus in patients with acute febrile illness from the Peruvian coast. BMC research notes **2020**; 13:67.

134. Martos-Benitez FD, Betancourt-Plaza I, Osorio-Carmenates I, et al. Neurological performance and clinical outcomes related to patients with Oropouche-associated Guillain-Barre syndrome. Journal of the peripheral nervous system **2025**; 30:e12683.

135. McGregor BL, Connelly CR, Kenney Joan L. Infection, dissemination, and transmission potential of North American Culex quinquefasciatus, Culex tarsalis, and Culicoides sonorensis for Oropouche virus. Viruses **2021**; 13:226.

136. Medlin S, Deardorff Eleanor R, Hanley Christopher S, et al. Serosurvey of selected arboviral pathogens in free-ranging, two-toed sloths (Choloepus hoffmanni) and three-toed sloths (Bradypus variegatus) in Costa Rica, 2005-07. Journal of wildlife diseases **2016**; 52:883–92.

137. Migueres M, Garnier C, Tremeaux P, et al. Oropouche Fever: An imported case underscoring the importance of clinical and epidemiological vigilance. Travel medicine and infectious disease **2025**; 66:102862.

138. Moreira HM, Sgorlon G, Queiroz Jackson AS, et al. Outbreak of Oropouche virus in frontier regions in western Amazon. Microbiology spectrum **2024**; 12:e0162923.

139. Moreli M, Aquino V, Figueiredo L. Identification of Simbu, California and Bunyamwera serogroup bunyaviruses by nested RT-PCR. Transactions of the Royal Society of Tropical Medicine and Hygiene **2001**; 95:108–13.

140. Moreli ML, Aquino Victor H, Cruz Ana Cecilia R, Figueiredo Luiz Tadeu M. Diagnosis of Oropouche virus infection by RT-nested-PCR. Journal of medical virology **2002**; 66:139–42.

141. Mores CN, Turell Michael J, Dyer J, Rossi Cindi A. Phylogenetic relationships among orthobunyaviruses isolated from mosquitoes captured in Peru. Vector borne and zoonotic diseases **2009**; 9:25–32.

142. Mourão MP, Bastos Michelle S, Gimaqu Joao Boscoe L, et al. Oropouche fever outbreak, Manaus, Brazil, 2007-2008. Emerging infectious diseases **2009**; 15:2063–4.

143. Muller V, Soares R, dos Santos-Junior N, et al. Phospholipase A2 Isolated from the Venom of Crotalus durissus terrificus Inactivates Dengue virus and Other Enveloped Viruses by Disrupting the Viral Envelope. PLoS ONE **2014**; 9:e112351.

144. Murillo JL, Cabral Aline D, Uehara M, et al. Nucleoprotein from the unique human infecting Orthobunyavirus of Simbu serogroup (Oropouche virus) forms higher order oligomers in complex with nucleic acids in vitro. Amino acids **2018**; 50:711–21.

145. Navarro J-C, Giambalvo D, Hernandez R, et al. Isolation of Madre de Dios virus (Orthobunyavirus; Bunyaviridae), an Oropouche virus species reassortant, from a monkey in Venezuela. The American journal of tropical medicine and hygiene **2016**; 95:328–38.

146. Naveca FG, Almeida Tatiana Amaral Pires d, Souza V, et al. Human outbreaks of a novel reassortant Oropouche virus in the Brazilian Amazon region. Nature medicine **2024**; 30:3509–21.

147. Naveca FG, Nascimento Valdinete A, Souza Victor C, de Figueiredo Regina MP. Human Orthobunyavirus infections, Tefe, Amazonas, Brazil. PLoS currents **2018**; 10.

148. Naveca FG, Nascimento Valdinete Alves d, Souza Victor Costa d, Nunes Bruno Tardelli D, Rodrigues Daniela Sueli G, Vasconcelos Pedro Fernando da C. Multiplexed reverse transcription real-time polymerase chain reaction for simultaneous detection of Mayaro, Oropouche, and Oropouche-like viruses. Memorias do Instituto Oswaldo Cruz **2017**; 112:510–3.

149. Naveca FG, Nascimento Valdinete Alves d, Souza Victor Costa d, et al. Development of CEL: a low-cost equipment for Loop-mediated Isothermal Amplification (LAMP) assays. Comunicação em Ciências da Saúde **2017**; 28:31–5.

150. Pauvolid-Correa A, Campos Z, Soares R, Nogueira Rita Maria R, Komar N. Neutralizing antibodies for orthobunyaviruses in Pantanal, Brazil. PLoS neglected tropical diseases **2017**; 11:e0006014.

151. Payne AF, Stout J, Dumoulin P, et al. Lack of competence of US mosquito species for circulating Oropouche virus. Emerging infectious diseases **2024**; 31:619–21.

152. Peiro-Mestres A, Riera E, Flores C, Navero-Castillejos J, Martinez MJ, Camprubi-Ferrer D. Emergence of Oropouche virus among international travelers: A growing concern in non-endemic areas. Enfermedades Infecciosas y Microbiologia Clinica **2025**:EIMC–2953.

153. Pereira-Silva JW, Rios-Velasquez Claudia M, Lima Gervilane Ribeiro d, et al. Distribution and diversity of mosquitoes and Oropouche-like virus infection rates in an Amazonian rural settlement. PloS one **2021**; 16:e0246932.

154. Perez YM, Ibanez Ariamys C, Diaz Zulema M, et al. First report of Culicoides paraensis (Goeldi, 1905) (Diptera: Ceratoponidae) in Cuba: A new challenge for public health. Parasite epidemiology and control **2025**; 29:e00423.

155. Perez-Restrepo LS, Knuese C, Vargas V, et al. Protocol for detection of Oropouche viruses from human serum. STAR protocols **2025**; 6:103805.

156. Phan TG, Del Valle Mendoza J, Sadeghi M, Altan E, Deng X, Delwart E. Sera of Peruvians with fever of unknown origins include viral nucleic acids from non-vertebrate hosts. Virus genes **2018**; 54:33–40.

157. Pimentel EP, Queiroz Jackson Alves da S, Ribeiro Jessiane R, et al. Identification of Mayaro virus genotype D in Rondonia, Brazil. The American journal of tropical medicine and hygiene **2024**; 110:557–60.

158. Pinheiro F, Pinheiro M, Bensabath G, Causey OR, Shope R. Epidemic of Oropouche virus in Belem. Revista do Serviço Especial de Saúde Pública **1962**; 12:15–23.

159. Pinheiro FP, Hoch AL, Gomes ML, Roberts DR. Oropouche virus. IV. Laboratory transmission by Culicoides paraensis. The American journal of tropical medicine and hygiene **1981**; 30:172–6.

160. Pinheiro FP, Rocha AG, Freitas RB, et al. Meningitis associated with infections due to Oropouche virus. Revista do Instituto de Medicina Tropical de São Paulo **1982**; 24:246–51.

161. Pinheiro FP, Rosa APATd, Rosa JFTd, Bensabath G. An outbreak of Oropouche virus disease in the vicinity of Santarem, Para, Brazil. Tropenmedizin und Parasitologie **1976**; 27:213–23.

162. Pinheiro FP, Travassos da Rosa AP, Gomes ML, LeDuc JW, Hoch AL. Transmission of Oropouche virus from man to hamster by the midge Culicoides paraensis. Science **1982**; 215:1251–3.

163. Pinto AK, Williams Graham D, Szretter Kristy J, et al. Human and murine IFIT1 proteins do not restrict infection of negative-sense RNA viruses of the Orthomyxoviridae, Bunyaviridae, and Filoviridae families. Journal of virology **2015**; 89:9465–76.

164. Pinto de Figueiredo RM, Thatcher Bedsy D, de Lima Mario L, Almeida Tania C, Alecrim Wilson D, Guerra Marcus Vinitius de F. Exanthematous diseases and the first epidemic of dengue to occur in Manaus, Amazonas State, Brazil, during 1998-1999. Revista da Sociedade Brasileira de Medicina Tropical **2004**; 37:476–9.

165. Pomari E, Matucci A, Accordini S, et al. ddPCR for the detection and absolute quantification of Oropouche virus. Viruses **2024**; 16:1426.

166. Portillo MT, Marwah A, Kraemer MUG, Thomas-Bachli A, Khan K, Bogoch II. Potential for international spread of Oropouche virus via commercial air travel. Journal of Travel Medicine **2024**; 31:taae128.

167. Proenca-Modena JL, Hyde Jennifer L, Sesti-Costa R, et al. Interferon-regulatory factor 5-dependent signaling restricts Orthobunyavirus dissemination to the central nervous system. Journal of virology **2016**; 90:189–205.

168. Proenca-Modena JL, Sesti-Costa R, Pinto Amelia K, et al. Oropouche virus infection and pathogenesis are restricted by MAVS, IRF-3, IRF-7, and type I interferon signaling pathways in nonmyeloid cells. Journal of virology **2015**; 89:4720–37.

169. Ramirez Pavon JA, Neves Nilvanei Aparecido da S, Silva Ludmilla Campos F, et al. Neurological infection by chikungunya and a triple Arbovirus co-infection in Mato Grosso, Central Western Brazil during 2019. Journal of clinical virology **2022**; 146:105056.

170. Rebello Moreira FR, Dutra JVR, de Carvalho AHB, et al. Oropouche virus genomic surveillance in Brazil. The Lancet Infectious Diseases **2024**; 24:E664–E6.

171. Rebello Moreira FR, Monteiro Fabio Luis L, de Menezes Mariane T, et al. Genomic evidence of Oropouche virus autochthonous circulation in a small district in the state of Rio de Janeiro, Brazil. Microbiology spectrum **2025**; 13:e0285024.

172. Restrepo-Lopez N, Silva-Ramos CR, Rodas JD, et al. Malaria, Dengue fever, and Leptospirosis in the Uraba Antioqueno region, Colombia: etiological and molecular characterization among patients with acute undifferentiated febrile illness. American Journal of Tropical Medicine and Hygiene **2025**; 112:403–13.

173. Ribeiro A, Mariene, Cornejo P, et al. Oropouche virus infects, persists and induces IFN response in human peripheral blood mononuclear cells as identified by RNA PrimeFlow TM and qRT-PCR assays. Viruses **2020**; 12:785.

174. Ribeiro Cruz AC, Prazeres Assis do Socorro Correa d, Gama Eliana C, et al. Serological survey for arboviruses in Juruti, Pará State, Brazil. Cadernos de Saúde Pública **2009**; 25:2517–23.

175. Rios-González CM, De Benedictis-Serrano Ginno A, Flores-Enríquez Jessica F, Chirino-Caicedo Alfonzo D. Knowledge, attitudes and practices about the oropouche, in medical students of Latin America, 2017. Revista del Instituto de Medicina Tropical **2017**; 12:14–22.

176. Rodrigues AH, Santos Rodrigo I, Arisi Gabriel M, et al. Oropouche virus experimental infection in the golden hamster (Mesocrisetus auratus). Virus research **2011**; 155:35–41.

177. Rojas A, Stittleburg V, Cardozo F, et al. Real-time RT-PCR for the detection and quantitation of Oropouche virus. Diagnostic microbiology and infectious disease **2020**; 96:114894.

178. Romero-Alvarez D, Escobar Luis E. Vegetation loss and the 2016 Oropouche fever outbreak in Peru. Memorias do Instituto Oswaldo Cruz **2017**; 112:292–8.

179. Romero-Alvarez D, Escobar Luis E, Auguste Albert J, Del Valle Sara Y, Manore Carrie A. Transmission risk of Oropouche fever across the Americas. Infectious diseases of poverty **2023**; 12.

180. Rosa AP, Rodrigues Sueli G, Nunes Márcio RT, Magalhäes Mioni TF, Rosa Jorge FST, Vasconcelos Pedro FC. Fever epidemic of oropouche virus in Serra Pelada, municipality of Curionópolis, Pará State, 1994. Revista da Sociedade Brasileira de Medicina Tropical **1996**; 29:537–41.

181. Rosato RR, Brand OM, Bagley LR. Retention of fluorescent antigenicity of virus-infected cells on spotslides under various conditions of storage. JOURNAL OF VIROLOGICAL METHODS **1982**; 5:279–84.

182. Saatkamp CJ, Rodrigues Luis Reginaldo R, Pereira Andrew Mairom N, et al. Mayaro virus detection in the western region of Para state, Brazil. Revista da Sociedade Brasileira de Medicina Tropical **2021**; 54:e0055–2020.

183. Saeed MF, Nunes M, Vasconcelos PF, et al. Diagnosis of Oropouche virus infection using a recombinant nucleocapsid protein-based enzyme immunoassay. Journal of clinical microbiology **2001**; 39:2445–52.

184. Saeed MF, Wang H, Nunes M, et al. Nucleotide sequences and phylogeny of the nucleocapsid gene of Oropouche virus. The Journal of general virology **2000**; 81:743–8.

185. Saeed MF, Wang H, Suderman M, et al. Jatobal virus is a reassortant containing the small RNA of Oropouche virus. Virus research **2001**; 77:25–30.

186. Saivish MV, Menezes Gabriela de L, da Silva Roosevelt A, et al. Acridones as promising drug candidates against Oropouche virus. Current research in microbial sciences **2024**; 6:100217.

187. Sanchez-Lerma L, Rojas-Gulloso A, Miranda J, et al. Unexpected arboviruses found in an epidemiological surveillance of acute tropical febrile syndrome in the department of Meta, Eastern Colombia. Journal of infection and public health **2024**; 17:102510.

188. Santos RI, Almeida Mariana FP, Paula Flavia E, et al. Experimental infection of suckling mice by subcutaneous inoculation with Oropouche virus. Virus research **2012**; 170:25–33.

189. Santos RI, Bueno-Junior Lezio S, Ruggiero Rafael N, et al. Spread of Oropouche virus into the central nervous system in mouse. Viruses **2014**; 6:3827–36.

190. Santos RI, Rodrigues Alcir H, Silva Maria L, et al. Oropouche virus entry into HeLa cells involves clathrin and requires endosomal acidification. Virus research **2008**; 138:139–43.

191. Scachetti GC, Forato J, Claro Ingra M, et al. Re-emergence of Oropouche virus between 2023 and 2024 in Brazil: an observational epidemiological study. The Lancet Infectious diseases **2025**; 25:166–75.

192. Schwarz MM, Price David A, Ganaie Safder S, et al. Oropouche orthobunyavirus infection is mediated by the cellular host factor Lrp1. Proceedings of the National Academy of Sciences of the United States of America **2022**; 119:e2204706119.

193. Silva J, J VJ, Lopes TRR, et al. Unprecedented Oropouche fever outbreak in Brazil: could the M segment-encoded proteins provide clues to possible insights? Journal of Medical Virology **2024**; 96:e70112.

194. Silva LB, Silva Laura Leone d, de Araujo Leonardo P, Silva Evandro N, Corsetti Patricia P, de Almeida Leonardo A. A computational approach for MHC-restricted multi-epitope vaccine design targeting Oropouche virus structural proteins. Acta tropica **2025**; 263:107575.

195. Silva-Caso W, Aguilar-Luis Miguel A, Palomares-Reyes C, et al. First outbreak of Oropouche Fever reported in a non-endemic western region of the Peruvian Amazon: Molecular diagnosis and clinical characteristics. International journal of infectious diseases **2019**; 83:139–44.

196. Silva-Nunes M, Malafronte R, Luz B, et al. The Acre Project: the epidemiology of malaria and arthropod-borne virus infections in a rural Amazonian population. Cadernos de Saude Publica **2006**; 22:1325–34.

197. Silva-Ramos CR, Gil-Mora J, Serna-Rivera Cristian C, et al. Etiological characterization of acute undifferentiated febrile illness in Apartado and Villeta municipalities, Colombia, during COVID-19 pandemic. Le infezioni in medicina **2023**; 31:517–32.

198. Smith GC, Francy DB. Laboratory studies of a Brazilian strain of Aedes albopictus as a potential vector of Mayaro and Oropouche viruses. Journal of the American Mosquito Control Association **1991**; 7:89–93.

199. Stubbs SH, Cornejo P, Marjorie, et al. Vesicular stomatitis virus chimeras expressing the Oropouche virus glycoproteins elicit protective immune responses in mice. mBio **2021**; 12:e0046321.

200. Tavares-Neto J, Freitas-Carvalho J, Nunes Marcio Roberto T, et al. Serologic survey for yellow fever and other arboviruses among inhabitants of Rio Branco, Brazil, before and three months after receiving the yellow fever 17D vaccine. Revista da Sociedade Brasileira de Medicina Tropical **2004**; 37:1–6.

201. Teixeira de Almeida AL, da Costa Igor Pinto S, Garcia Maycon Douglas do N, et al. Oropouche virus: isolation and ultrastructural characterization from a human case sample from Rio de Janeiro, Brazil, using an in vitro system. Viruses **2025**; 17.

202. Teixeira Nunes MR, Barbosa Taciana Fernandes S, Casseb Livia Medeiros N, et al. Arbovirus eco-epidemiology in the area affected by the Cuiaba-Santarem Highway (BR-163), Para State, Brazil. Cadernos de Saúde Pública **2009**; 25:2583–602.

203. Teixeira Nunes MR, de Souza William M, Savji N, et al. Oropouche orthobunyavirus: Genetic characterization of full-length genomes and development of molecular methods to discriminate natural reassortments. Infection, genetics and evolution **2019**; 68:16–22.

204. Teixeira Nunes MR, Martins Livia C, Rodrigues Sueli G, et al. Oropouche virus isolation, southeast Brazil. Emerging infectious diseases **2005**; 11:1610–3.

205. Tilston-Lunel NL, Acrani Gustavo O, Randall Richard E, Elliott Richard M. Generation of recombinant Oropouche viruses lacking the nonstructural protein NSm or NSs. Journal of virology **2015**; 90:2616–27.

206. Tilston-Lunel NL, Hughes J, Acrani Gustavo O, et al. Genetic analysis of members of the species Oropouche virus and identification of a novel M segment sequence. The Journal of general virology **2015**; 96:1636–50.

207. Tilston-Lunel NL, Shi X, Elliott Richard M, Acrani Gustavo O. The potential for reassortment between Oropouche and Schmallenberg Orthobunyaviruses. Viruses **2017**; 9:220.

208. Turell MJ, Gozalo Alfonso S, Guevara C, et al. Lack of evidence of sylvatic transmission of dengue viruses in the Amazon rainforest near Iquitos, Peru. Vector borne and zoonotic diseases **2019**; 19:685–9.

209. Usuga J, Limonta D, Perez-Restrepo Laura S, et al. Co-Circulation of 2 Oropouche Virus Lineages, Amazon Basin, Colombia, 2024. Emerging infectious diseases **2024**; 30:2375–80.

210. van Tongeren HA. Occurrence of arboviruses belonging to the C-, Bunyamwera and Guama groups, and of Oropouche, Junin, Tacaiuma and Kwatta viruses in man in the province of Brokopondo, Surinam: a serological survey. Tropical and geographical medicine **1967**; 19:309–25.

211. Varela M, Piras Ilaria M, Mullan C, et al. Sensitivity to BST-2 restriction correlates with Orthobunyavirus host range. Virology **2017**; 509:121–30.

212. Vasconcelos HB, Azevedo Raimunda SS, Casseb Samir M, et al. Oropouche fever epidemic in Northern Brazil: epidemiology and molecular characterization of isolates. Journal of clinical virology **2009**; 44:129–33.

213. Vasconcelos HB, Nunes Marcio RT, Casseb Livia MN, et al. Molecular epidemiology of Oropouche virus, Brazil. Emerging infectious diseases **2011**; 17:800–6.

214. Vernal S, Martini Camila CR, da Fonseca Benedito AL. Oropouche virus-associated aseptic meningoencephalitis, southeastern Brazil. Emerging infectious diseases **2019**; 25:380–2.

215. Viana Geddes VE, Brustolini Otavio Jose B, Cavalcante Liliane Tavares de F, et al. Common dysregulation of innate immunity pathways in human primary astrocytes infected with Chikungunya, Mayaro, Oropouche, and Zika viruses. Frontiers in cellular and infection microbiology **2021**; 11:641261.

216. Viana Geddes VE, de Oliveira Anibal S, Tanuri A, Arruda E, Ribeiro-Alves M, Aguiar Renato S. MicroRNA and cellular targets profiling reveal miR-217 and miR-576-3p as proviral factors during Oropouche infection. PLoS neglected tropical diseases **2018**; 12:e0006508.

217. Vieira C, da Silva DJF, Barreto ES, et al. Detection of Mayaro virus infections during a dengue outbreak in Mato Grosso, Brazil. ACTA TROPICA **2015**; 147:12–6.

218. Vita S, Colavita F, Maffongelli G, et al. Viral shedding in saliva, axillary, rectal and vaginal swabs of an imported case of Dengue - Oropouche virus Co-infection. Travel medicine and infectious disease **2025**; 66:102854.

219. Walsh CS, Cardenas Jenny C, Gutierrez-Silva Lady Y, et al. Investigating Oropouche as a possible etiology for febrile illness in a clinical cohort from Colombia, 2014-2015. The American journal of tropical medicine and hygiene **2025**; 113:102–5.

220. Wang H, Beasley DW, Li L, Holbrook MR, Barrett AD. Nucleotide sequence and deduced amino acid sequence of the medium RNA segment of Oropouche, a Simbu serogroup virus: comparison with the middle RNA of Bunyamwera and California serogroup viruses. Virus research **2001**; 73:153–62.

221. Watts DM, Lavera V, Callahan J, et al. Venezuelan equine encephalitis and Oropouche virus infections among Peruvian army troops in the Amazon region of Peru. The American journal of tropical medicine and hygiene **1997**; 56:661–7.

222. Watts DM, Phillips I, Callahan JD, Griebenow W, Hyams KC, Hayes CG. Oropouche virus transmission in the Amazon River basin of Peru. The American journal of tropical medicine and hygiene **1997**; 56:148–52.

223. Watts DM, Russell Kevin L, Wooster Mark T, et al. Etiologies of acute undifferentiated febrile illnesses in and near Iquitos from 1993 to 1999 in the Amazon river basin of Peru. The American journal of tropical medicine and hygiene **2022**; 107:1114–28.

224. Weidmann M, Rudaz V, Nunes Marcio RT, Vasconcelos Pedro FC, Hufert Frank T. Rapid detection of human pathogenic orthobunyaviruses. Journal of clinical microbiology **2003**; 41:3299–305.

225. Wise EL, Marquez S, Mellors J, et al. Oropouche virus cases identified in Ecuador using an optimised qRT-PCR informed by metagenomic sequencing. PLoS neglected tropical diseases **2020**; 14:e0007897.

226. Wise EL, Pullan Steven T, Marquez S, et al. Isolation of Oropouche virus from febrile patient, Ecuador. Emerging infectious diseases **2018**; 24:935–7.

227. Wongsurawat T, Jenjaroenpun P, Taylor MK, et al. Rapid sequencing of multiple RNA viruses in their native form. Frontiers in Microbiology **2019**; 10:260.

228. Arantes I, Bruycker-Nogueira Fernanda d, de Oliveira C, et al. Sustained Oropouche virus transmission in Rio de Janeiro's Atlantic forest: genomic evidence over a two-year period, **2025**.

229. Chuquimia Valdez JA, Arantes I, Martínez Sebastián S, et al. Genomic characterization, origin, and local transmission of Oropouche Virus in Bolivia in 2024, **2024**.

230. Connors KA, Pedlow Maris R, Frey Zachary D, et al. Characterization of neural infection by Oropouche orthobunyavirus, **2024**.

231. da Costa F, Cavalcanti A, Erbisti R, et al. Introduction and Spatial-Temporal Distribution of Oropouche Virus in Rio De Janeiro State, Brazil, **2024**.

232. dos Santos FM, de Melo ES, de Lima GB, et al. Oropouche virus importation in Southern Brazil and emerging concern calling for enhanced public health surveillance, **2025**.

233. Gravier R, Perez MM, Alvarez M, et al. Countrywide spread and spatiotemporal diffusion dynamics of Oropouche virus in Cuba, 2024, **2025**.

234. Hernandez S, Cardozo F, Myers DR, Rojas A, Waggoner JJ. Simple and economical RNA extraction and storage packets for viral detection from serum or plasma, **2022**.

235. Megli C, Zack RK, McGaughey JJ, et al. Oropouche virus infects human placenta explants and trophoblast organoids, **2024**.

236. Merchant MK, de Paula Souza J, Abdelkarim S, et al. Protein-based tools for the detection and characterisation of Oropouche virus infection. medRxiv, **2025**.

237. Olortegui MP, Schiaffino F, Penataro Y, et al. Genomic Epidemiology of 2023-2024 Oropouche Outbreak in Iquitos, Peru reveals independent origin from a concurrent outbreak in Brazil, **2024**.

238. Pilgrim J, Sy Victoria E, Multeau Q, et al. Lack of vector competence in UK Culex pipiens molestus for Oropouche virus, **2025**.

239. Poongavanan J, Dunaiski M, D'or G, et al. Spatiotemporal disease suitability prediction for Oropouche virus and the role of vectors across the Americas, **2025**.

240. Requena-Zuniga E, Palomino-Salcedo M, Garcia-Mendoza MP, et al. First detection of Oropouche virus in Culicoides insignis in the Ucayali region, Peru: evidence of a possible new vector, **2024**.

241. Tegally H, Dellicour S, Poongavanan J, et al. Dynamics and ecology of a multi-stage expansion of Oropouche virus in Brazil. medRxiv **2024**.

242. Xiong J, Wang Y, Hou J, et al. The elusive birth of Oropouche and Oropouche-like viruses: a genomic analysis, **2024**.

243. European Centre for Disease Prevention and Control. Communicable disease threats report Week 23, 1−7 June 2024, **2024**.

244. European Centre for Disease Prevention and Control. Communicable disease threats report Week 25, 15 - 21 June 2024, **2024**.

245. European Centre for Disease Prevention and Control. Communicable disease threats report Week 28, 6–12 July 2024, **2024**.

246. European Centre for Disease Prevention and Control. Communicable disease threats report Week 30, 20 - 26 July 2024, **2024**.

247. European Centre for Disease Prevention and Control. Threat assessment brief. Oropouche virus disease cases imported into the European Union 9 August 2024, **2024**.

248. Ghidoni C, Hollenstein N, Cusini A. Oropouche virus infection after travelling to Cuba. Praxis **2025**; 114:123–6.

249. Ministry of Health Epidemiological Division Brazil. Dengue outbreak in Boa Vista, Roraima Territory, Brazil. Epidemiological Bulletin **1982**; 3:5–7.

250. Morrison A, White Jennifer L, Hughes Holly R, et al. Oropouche virus disease among U.S. travelers - United States, 2024. Morbidity and mortality weekly report **2024**; 73:769–73.

251. Pan American Health Organization. Jungle yellow fever in Goias, Brazil. PAHO Epidemiological Bulletin. Vol. 1, **1980**:2057–9.

252. Pan American Health Organization. Epidemiological Alert, Outbreak of Oropouche Fever (22 June 2010), **2010**.

253. Pan American Health Organization. Epidemiological Alert Oropouche in the Region of the Americas 1 August 2024, **2024**.

254. Pan American Health Organization. Epidemiological Alert Oropouche in the Region of the Americas 9 May 2024, **2024**.

255. Pan American Health Organization. Epidemiological Alert Oropouche in the Americas Region 13 December 2024, **2024**.

256. Pan American Health Organization. Epidemiological Alert Oropouche in the Region of the Americas 2 February 2024, **2024**.

257. Pan American Health Organization. Epidemiological Alert Oropouche in the Region of the Americas: vertical transmission event under investigation in Brazil 17 July 2024, **2024**.

258. Pan American Health Organization. Epidemiological Update Oropouche in the Americas Region 15 October 2024, **2024**.

259. Pan American Health Organization. Epidemiological Update Oropouche in the Americas Region 6 September 2024, **2024**.

260. Pan American Health Organization. Epidemiological Update Oropouche in the Region of the Americas 6 March 2024, **2024**.

261. Pan American Health Organization. Epidemiological Update Oropouche in the Region of the Americas 12 April 2024, **2024**.

262. Pan American Health Organization. Public Health Risk Assessment related to Oropouche Virus (OROV) in the Region of the Americas 3 August 2024, **2024**.

263. Pan American Health Organization. Public Health Risk Assessment related to Oropouche Virus (OROV) in the Region of the Americas 9 February 2024, **2024**.

264. Pan American Health Organization. Epidemiological Update Oropouche in the Americas Region 11 February 2025, **2025**.

265. World Health Organization. Oropouche virus disease - Peru. Available at: https://www.who.int/emergencies/disease-outbreak-news/item/03-june-2016-oropouche-peru-en.

266. World Health Organization. Oropouche virus disease - French Guiana. Available at: https://www.who.int/emergencies/disease-outbreak-news/item/oropouche-virus-disease---french-guiana-france.

267. World Health Organization. Oropouche virus disease - Cuba. Available at: https://www.who.int/emergencies/disease-outbreak-news/item/2024-DON521.

268. World Health Organization. Oropouche virus disease - Region of the Americas. August. Available at: https://www.who.int/emergencies/disease-outbreak-news/item/2024-DON530.

269. World Health Organization. Oropouche virus disease - Region of the Americas. December. Available at: https://www.who.int/emergencies/disease-outbreak-news/item/2024-DON545.

**Supplementary material 4. List of excluded studies**

| **Author (year)** | **Title** |
| --- | --- |
| **Wrong study design (n=136)** | |
| Akingbola (2024) | The rising concern of Oropouche fever: a call for enhanced surveillance and research in emerging arboviral threats |
| Al-Tawfiq (2024) | The emergence of Oropouche virus in Cuba - A wake-up call for global health |
| Alencar (2008) | Potencialidades do Aedes albopictus como vetor de arboviroses no Brasil: um desafio para a atenção primária |
| Ali (2025) | Risk of Oropouche virus importation to Southeast Asia via international travellers |
| Anon (2024) | Oropouche fever, the mysterious threat |
| Atamari-Anahui (2018) | La implicancia del reordenamiento genético en el diagnóstico y la epidemiología del virus Oropuche en el Perú |
| Atamari-Anahui (2018) | The implication of genetic rearrangement in the diagnosis and epidemiology of Oropuche virus in Peru |
| Barcante (2024) | Surge in Oropouche fever: the tip of the iceberg in a new public health challenge in Brazil |
| Barcante (2025) | The growing challenge of arboviruses in Latin America: Dengue and Oropouche in focus |
| Bertolino (2024) | Oropouche virus infection: What internal medicine physicians should know |
| Bhattacharya (2025) | Possibility of invasion of Oropouche virus (OROV) in Asia: a real-time assessment is an imperative necessity |
| Branda (2024) | Oropouche virus presenting in Italy after travel to Cuba |
| Brickley (2024) | Preparing for the rapid research response to the possible vertical transmission of Oropouche virus: lessons from a decade of congenital Zika research |
| Brisse (2025) | Oropuche virus-an emerging pathogen with escalating risk for outbreaks of human infection |
| Cabada (2024) | Establishment of a multisite umbrella cohort study protocol to describe the epidemiology and aetiologies of acute undifferentiated febrile illness in Latin America |
| Cain (2024) | Oropouche virus: Understanding ‘sloth fever’ disease dynamics and novel intervention strategies against this emerging neglected tropical disease |
| Capobianchi (2024) | Potential risks of Oropouche virus importation into Europe |
| Castaño (2025) | Enfermedades re-emergentes y emergentes |
| Castilletti (2024) | Author's response: Oropouche virus risk for European travellers to Cuba: an emerging public health concern |
| Castilletti (2024) | First diagnoses of Oropouche virus in Europe: how can we strengthen communication and preparedness globally? |
| CDC (2024) | Increased Oropouche virus activity and associated risk to travelers |
| CDC (2024) | Response to Oropouche virus disease cases in U.S. states and territories in the Americas |
| CDC (2025) | 2024 Oropouche outbreak |
| CDC (2025) | Clinical overview of Oropouche virus disease |
| CDC (2025) | Interim clinical considerations for pregnant women with confirmed or probable Oropouche virus disease |
| CDC (2025) | Interim guidance for evaluating and managing infants born to pregnant women with confirmed or probable Oropouche virus disease |
| CDC (2025) | Updated interim guidance for health departments on testing and reporting for Oropouche virus disease |
| CDC (2025) | Updated interim guidance for health departments on testing and reporting for Oropouche virus disease |
| CDC (2025) | Clinical overview of Oropouche virus disease |
| Ceccarelli (2025) | Oropouche virus infection: Differential clinical outcomes and emerging global concerns of vertical transmission and fatal cases |
| Culquichicon (2017) | Bibliometric analysis of Oropouche research: impact on the surveillance of emerging arboviruses in Latin America |
| Dashraath (2025) | Vertical transmission potential of Oropouche virus infection in human pregnancies |
| de Souza (2024) | ICTV Virus Taxonomy Profile: Peribunyaviridae 2024 |
| Desai (2024) | Oropouche virus: A re-emerging arbovirus of clinical significance (doi: 10.1016/j.ijregi.2024.100456) |
| Desai (2024) | Oropouche virus: A re-emerging arbovirus of clinical significance (doi: 10.1016/j.ijid.2024.107251) |
| Dinh (2025) | Oropouche virus |
| Diniz (2024) | Oropouche fever in Brazil: When the time is now |
| Diniz (2025) | Additional lessons to prepare for rapid research response to possible vertical transmission of Oropouche virus in Brazil |
| Dobler (1996) | Arboviruses causing neurological disorders in the central nervous system |
| Douglas (2024) | The silent invaders: Oropouche and Melao viruses, causes of increased public health risks for the Americas |
| Dutra (2017) | The re-emerging arboviral threat: Hidden enemies: The emergence of obscure arboviral diseases, and the potential use of Wolbachia in their control |
| Edridge (2020) | Emerging orthobunyaviruses associated with CNS disease |
| Elliott (1997) | Emerging viruses: the Bunyaviridae |
| Escobedo (2024) | Oropouche fever rears its head in Cuba: What lies beneath the surface? |
| Figueiredo (1999) | Vírus brasileiros da família Bunyaviridae |
| Figueiredo (2007) | Emergent arboviruses in Brazil |
| Figueiredo (2014) | Saint Louis encephalitis virus and other arboviruses in the differential diagnosis for dengue |
| Figueiredo (2019) | Human urban arboviruses can infect wild animals and jump to sylvatic maintenance cycles in South America |
| Files (2022) | Baseline mapping of Oropouche virology, epidemiology, therapeutics, and vaccine research and development |
| Garima (2025) | Silent risk: the vertical transmission of Oropouche virus |
| Giovanetti (2024) | Letter to the editor: Oropouche virus risk for European travellers to Cuba: an emerging public health concern |
| Giovanetti (2025) | Oropouche virus and the urgent need for global surveillance |
| Goncalves (2024) | Oropouche Fever and its Current Context in Brazil |
| Gonzalez-Quevedo (2025) | Oropouche virus - another antecedent event for Guillain-Barre syndrome? |
| Gordon (1968) | Profilaxia das doencas transmissíveis |
| Hartman (2023) | Bunyavirales: Scientific Gaps and Prototype Pathogens for a Large and Diverse Group of Zoonotic Viruses |
| Hassan (2025) | Arboviral infections and pregnancy: An overlooked public health challenge |
| Hollidge (2010) | Arboviral encephalitides: transmission, emergence, and pathogenesis |
| Hopkins (2013) | Bunyaviral cap-snatching vs. decapping: Recycling cell cycle mRNAs |
| Hui (2024) | Summary of global surveillance data of infectious diseases in July 2024 |
| Huits (2024) | New insights into Oropouche: expanding geographic spread, mortality, vertical transmission, and birth defects |
| Jing (2025) | Risk assessment of global infectious disease events in December 2024 |
| Lavergne (2024) | Oroupouche virus: towards a future emergence? |
| LeDuc (1989) | Epidemiology of hemorrhagic fever viruses |
| Li (2024) | Innate immune response against vector-borne bunyavirus infection and viral countermeasures |
| Liu (2024) | Oropouche fever, a potential threat to global health? |
| Liu (2024) | Epidemiological and clinical overview of the 2024 Oropouche virus disease outbreaks, an emerging/re-emerging neurotropic arboviral disease and global public health threat |
| Lorenz (2024) | Brazil reports an increased incidence of Oropouche and Mayaro fever in the amazon region |
| Makarov (2016) | Zoopathogenic Orthobuniaviruses (Orthobunyavirus, Bunyaviridae) |
| Martins-Filho (2024) | Oropouche fever: reports of vertical transmission and deaths in Brazil |
| Martins-Filho (2024) | The underdiagnosed threat of Oropouche fever amidst dengue epidemics in Brazil |
| Mattar (2016) | Olympic Games Rio 2016 and the uninvited viruses: Potential consequences for Europe and North America |
| Mattar (2025) | The next arbovirus epidemic in Latin America and beyond: A question of when, not if - Mayaro, Oropouche, Usutu or Disease X? |
| McQuillan (2025) | Oropouche virus in Panama |
| Mira (2025) | Oropouche Virus: The Next (Re)Emerging Arboviral Threat? |
| Miranda-Filho (2024) | Preparing for the rapid research response to the possible vertical transmission of Oropouche virus: lessons from a decade of congenital Zika research |
| Mohapatra (2024) | Surging Oropouche virus (OROV) cases in the Americas: A public health challenge |
| Moreira-Soto (2025) | Transfusion-transmitted infections: risks and mitigation strategies for Oropouche virus and other emerging arboviruses in Latin America and the Caribbean |
| Mourao (2015) | Arboviral diseases in the Western Brazilian Amazon: a perspective and analysis from a tertiary health and research center in Manaus, State of Amazonas |
| Nunes (2007) | A febre do Oropouche: uma revisão dos aspectos epidemiológicos e moleculares na Amazônia brasileira |
| Okesanya (2025) | Addressing the emerging threat of Oropouche virus: implications and public health responses for healthcare systems |
| Organización Panamericana de la Salud (2016) | Instrumento para el diagnóstico y la atención a pacientes con sospecha de arbovirosis |
| Overturf (2009) | World arboviruses: the Bunyaviridae |
| PAHO (2024) | Recommendations for the Detection and Surveillance of Oropouche in possible cases of vertical infection, congenital malformation, or fetal death July 17, 2024 |
| PAHO (1982) | Oropouche fever in Brasil |
| PAHO (1982) | Dengue outbreak in Boa Vista, Roraima territory, Brazil |
| PAHO (1983) | Public health impact of arthropod- and rodent-borne viral diseases in the Americas |
| PAHO (2023) | Recommendations for laboratory detection and diagnosis of arbovirus infections in the region of the Americas |
| PAHO (2024) | Interim guidance on entomological surveillance and prevention measures for oropouche virus vectors |
| PAHO (2025) | Oropouche virus research agenda January 2025. Development of a research agenda for the characterization of Oropouche virus and its public health implications |
| Pareek (2024) | Emerging risk of sexual transmission of Oropouche virus |
| Pastula (2024) | Oropouche Virus: An Emerging Neuroinvasive Arbovirus |
| Patel (2025) | The Oropouche virus (OROV) and need of advanced diagnostics to mitigate it |
| Pereira (2025) | Oropouche virus disease-region of the Americas |
| Pinheiro (1980) | Arboviruses in the Amazon region, Brazil |
| Pinheiro (1997) | Febre por Oropouche |
| Policastro (2024) | Oropouche virus in Brazil: Assessing the risks and challenges for transfusion medicine |
| Porwal (2025) | Mysterious Oropouche virus: Transmission, symptoms, and control |
| Postigo-Hidalgo (2024) | Probing Oropouche fever ecology beyond the Amazon |
| Postigo-Hidalgo (2025) | Probing Oropouche fever ecology beyond the Amazon |
| Ribas (2025) | Oropouche Virus (OROV): Expanding Threats, Shifting Patterns, and the Urgent Need for Collaborative Research in Latin America |
| Rodriguez-Morales (2017) | Mayaro, Oropouche and Venezuelan Equine Encephalitis viruses: Following in the footsteps of Zika? |
| Rodriguez-Morales (2025) | Reemergence of Oropouche virus infection in Venezuela, 2025 |
| Romero (2006) | Diagnosis of viral encephalitides: zoonotic-associated viruses |
| Romero-Alvarez (2018) | Oropouche fever, an emergent disease from the Americas |
| Romero-Alvarez (2025) | The foretold Oropouche fever epidemic |
| Sah (2024) | Oropouche fever outbreak in Brazil: an emerging concern in Latin America |
| Samara (2024) | Potential vertical transmission of Oropouche virus during the current outbreak |
| Sanabria-Rojas (2015) | Clinical trials in neglected infectious diseases in the context of globalization: an ethical and human rights approach |
| Sansone (2024) | Prevent the spread of Oropouche fever in Brazil |
| Sansone (2025) | The Oropouche fever in Latin America: a hidden threat and a possible cause of microcephaly due to vertical transmission |
| Schwartz (2024) | Oropouche Virus (OROV) in Pregnancy: An Emerging Cause of Placental and Fetal Infection Associated with Stillbirth and Microcephaly following Vertical Transmission |
| Schwartz (2025) | Novel Reassortants of Oropouche Virus (OROV) Are Causing Maternal-Fetal Infection During Pregnancy, Stillbirth, Congenital Microcephaly and Malformation Syndromes |
| Schwartz (2025) | A potential mechanism of transplacental transmission of Oropouche virus in pregnancy |
| Sciancalepore (2022) | Presence and Multi-Species Spatial Distribution of Oropouche Virus in Brazil within the One Health Framework |
| Sick (2019) | *Culicoides* Biting Midges-Underestimated Vectors for Arboviruses of Public Health and Veterinary Importance |
| Silva-Caso (2025) | Oropouche virus fever |
| Singhal (2024) | Rising threat of Oropouche virus transmission from mother to child: An urgent call for action |
| Soldan (2005) | Emerging infectious diseases: the Bunyaviridae |
| Souza (2024) | Oropouche fever and pregnancy |
| Srivastava (2024) | Pregnancy Loss, Oropouche Virus and the Lessons from Pernambuco, Brazil |
| Sun (2025) | Risk assessment of global infectious disease events in January 2025 |
| ter Horst (2019) | Structural and functional similarities in bunyaviruses: Perspectives for pan-bunya antivirals |
| Tesh (1994) | The emerging epidemiology of Venezuelan hemorrhagic fever and Oropouche fever in tropical South America |
| Toledo (2024) | Preparedness for emerging epidemic threats: detection of Oropouche circulation in Cuba |
| Travassos (1992) | Arboviruses in Serra Norte, Carajas region, Para, Brazil |
| Ukoaka (2024) | Updated WHO list of emerging pathogens for a potential future pandemic: Implications for public health and global preparedness |
| Valentine (2019) | Sylvatic cycles of arboviruses in non-human primates |
| Valero (2017) | Oropouche Virus: what is it and how it is transmitted? |
| Vasconcelos (1992) | Clinical and ecoepidemiological situation of human arboviruses in Brazilian Amazonia |
| Vasconcelos (2016) | Emergence of Human Arboviral Diseases in the Americas, 2000-2016 |
| Wermelinger (2023) | The conflict mediation potential to control arboviruses vectors in Brazilian slums |
| WHO (2024) | Oropouche virus disease |
| Yoosuf (2025) | Concerns and implications of Oropouche virus infection during pregnancy: A call for further research and public health action |
| Yvon (2024) | The genome of a bunyavirus can only be defined at the scale of the viral population |
| Zhang (2009) | Advances in Bunyaviridae viruses that cause human diseases |
| **Not oropouche (n=72)** | |
| Alencar (2011) | New report on the bionomics of *Coquillettidia venezuelensis* in temporary breeding sites (Diptera: Culicidae) |
| Anonymous (1993) | Emerging infectious diseases |
| Augot (2017) | Association between host species choice and morphological characters of main sensory structures of *Culicoides* in the Palaeartic region |
| Ayala (2022) | Redescription of immature stages of *Culicoides paraensis* (Goeldi) (Diptera: Ceratopogonidae), vector of the Oropouche virus |
| Ayers (2022) | Replication Kinetics of a Candidate Live-Attenuated Vaccine for Cache Valley Virus in *Aedes albopictus* |
| Beaty (1981) | Molecular basis of bunyavirus transmission by mosquitoes: role of the middle-sized RNA segment |
| Berezina (1983) | Effect of ribavirin on bunyavirus reproduction in cell culture and in an experiment on white mice |
| Binder (2019) | Molecular survey of flaviviruses and orthobunyaviruses in *Amblyomma* spp. ticks collected in Minas Gerais, Brazil |
| Blitvich (2012) | Orthobunyavirus antibodies in humans, Yucatan Peninsula, Mexico |
| Brenner (2016) | What can Akabane disease teach us about other arboviral diseases |
| Brenner (2019) | Serological evidence suggests that several Simbu serogroup viruses circulated in Israel |
| Camarao (2019) | Development and analytical validation of a group-specific RT-qPCR assay for the detection of the Simbu serogroup orthobunyaviruses |
| Cardoso (2010) | New records and epidemiological potential of certain species of mosquito (Diptera, Culicidae) in the State of Rio Grande do Sul, Brazil |
| Cooper (2025) | Laboratory and field assays indicate that a widespread no-see-um, *Culicoides furens* (Poey) is susceptible to permethrin |
| Damasceno (2015) | Mosquito (Diptera: Culicidae) fauna in parks in greater São Paulo, Brazil |
| de Brito (2007) | Caraparu virus (group C Orthobunyavirus): sequencing and phylogenetic analysis based on the conserved region 3 of the RNA polymerase gene |
| de Oliveira (2016) | Study of mortality from infectious diseases in Brazil from 2005 to 2010: risks involved in handling corpses |
| Dietrich (2017) | The Antiviral RNAi Response in Vector and Non-vector Cells against Orthobunyaviruses |
| do Carmo Silva (2023) | Advancing Chikungunya Diagnosis: A Cost-Effective and Rapid Visual employing Loop-mediated isothermal reaction |
| Farias (2020) | Diversity of biting midges *Culicoides* (Diptera: Ceratopogonidae), potential vectors of disease, in different environments in an Amazonian rural settlement, Brazil |
| Felippe-Bauer (2008) | *Culicoides paraensis* (Diptera: Ceratopogonidae) infestations in cities of the Itapocu River Valley, southern Brazil |
| Frey (2024) | Lrp1 facilitates infection of neurons by Jamestown Canyon virus |
| Gomes (2025) | Congenital anomalies in Brazil, 2010 to 2022 |
| Gutiérrez (1975) | Aislamiento de virus del grupo Anopheles A en Guatemala |
| Gutiérrez (1975) | Aislamiento del virus Mermet en Guatemala |
| Gutiérrez-Vera (2021) | Seroprevalence of arboviruses in Ecuador: Implications for improved surveillance |
| Hang (2012) | Random amplification and pyrosequencing for identification of novel viral genome sequences |
| Hart (2009) | Role of the NSs protein in the zoonotic capacity of Orthobunyaviruses |
| Hoch (1986) | Breeding sites of *Culicoides paraensis* and options for control by environmental management |
| Hoffmann (2012) | Novel orthobunyavirus in Cattle, Europe, 2011 |
| Hudson (1986) | Annotated list of the Ceratopogonidae (Diptera) of Suriname |
| Huerta (2024) | New records of *Culicoides (haematomyidium) paraensis* and a key to adult *Culicoides* from Yucatan peninsula, Mexico |
| Jentes (2010) | Acute arboviral infections in Guinea, West Africa, 2006 |
| Kato (2016) | Bovine Arboviruses in *Culicoides* Biting Midges and Sentinel Cattle in Southern Japan from 2003 to 2013 |
| Le Lay-Rogues (1984) | Serologic survey on arbovirus infections in patients presenting with uveitis |
| Li (2011) | Fever with thrombocytopenia associated with a novel bunyavirus in China |
| Lin (2016) | A rare fatal case of a novel bunyavirus-associated hemophagocytic lymphohistiocytosis |
| Magiorkinis (2011) | A novel bunyavirus in China |
| Martin (2000) | Standardization of immunoglobulin M capture enzyme-linked immunosorbent assays for routine diagnosis of arboviral infections |
| Martínez (2021) | Training strategy for the diagnosis and management of arbovirosis in Cienfuegos |
| Méndez (2009) | Arbovirus surveillance in the Department of Piura. Peru - 2008 |
| Mendez-Andrade (2024) | Habitat anthropization and seasonality affect the assemblage and diversity of *Culicoides* in central Veracruz, Mexico |
| Mercer (2003) | Biting rates and developmental substrates for biting midges (Diptera: Ceratopogonidae) in Iquitos, Peru |
| Mercer (2005) | Changes in relative species compositions of biting midges (Diptera: Ceratopogonidae) and an outbreak of Oropouche virus in Iquitos, Peru |
| Mettler (1982) | Virus inoculation in mice bearing Ehrlich ascitic tumors - antigen production and tumor-regression |
| Mitchell (1990) | Vector competence of Aedes albopictus for a newly recognized Bunyavirus from mosquitoes collected in Potosi, Missouri |
| Muttis (2023) | Mosquito diversity from the Urutaú Natural Reserve (Misiones, Argentina) |
| Odhiambo (2015) | Orthobunyavirus antibodies among humans in selected parts of the Rift Valley and northeastern Kenya |
| Ojha (2024) | N4-Hydroxycytidine/molnupiravir inhibits RNA virus-induced encephalitis by producing less fit mutated viruses |
| Organização Pan-Americana da Saúde (2025) | Documento operacional de identificação de *Culicoides* Latreille (Diptera: Ceratopogonidae) |
| Pinheiro (1977) | Public health hazards among workers along the Trans Amazon highway |
| Reis (2025) | Culicidae Fauna (Diptera: Culicomorpha) of the Quilombola Community of Abacatal, Ananindeua, Pará, in the Brazilian Amazon |
| Ricapa-Antay (2018) | Molecular detection and clinical characteristics of Bartonella bacilliformis, *Leptospira* spp., and *Rickettsia* spp. in the Southeastern Peruvian Amazon basin |
| Roberts (1981) | Oropouche virus. III. Entomological observations from three epidemics in Para, Brazil, 1975 |
| Roberts (1981) | Programa multidisciplinario de vigilancia de las enfermedades infecciosas en zonas colindantes con la Carretera Transamazónica en Brasil. IV. Estudio entomológico |
| Ronderos (2003) | Diversity of biting midges of the genus *Culicoides* Latreille (Diptera: Ceratopogonidae) in the area of the Yacyreta Dam Lake between Argentina and Paraguay |
| Sankhe (2025) | Genomic Insights into Neglected Orthobunyaviruses: Molecular Characterization and Phylogenetic Analysis |
| Santamaria (2024) | *Culicoides* Latreille (Diptera, Ceratopogonidae) of Colombia: records from the collection of insects of medical importance from National Institute of Health |
| Santana (2010) | Concurrent Dengue and malaria in the Amazon region |
| Scherer (1975) | The first isolations of eastern encephalitis, group C, and Guama group arboviruses from the Peruvian Amazon region of western South America |
| Scherer (1975) | Los primeros aislamientos de arbovirus de encefalitis del este y de grupos C y Guama en la región amazónica del Perú |
| Schneider (2024) | Skin muscle is the initial site of viral replication for arboviral bunyavirus infection |
| Schnettler (2013) | RNA interference targets arbovirus replication in *Culicoides* cells |
| Takahashi (1968) | Aino virus, a new member of Simbu group of an arbovirus front mosquitoes in Japan |
| Temmam (2016) | Characterization of Viral Communities of Biting Midges and Identification of Novel Thogotovirus Species and Rhabdovirus Genus |
| Van der Waals (1986) | Post-encephalitic epilepsy and arbovirus infections in an isolated rainforest area of central Liberia |
| Velásquez (2014) | Bionomics, ecology and medical importance of *Coquillettidea (Rhynchotaenia) venezuelensis* Theobald, 1912 (Diptera: Culicidae) |
| Vieira (2022) | Land-use effects on mosquito biodiversity and potential arbovirus emergence in the Southern Amazon, Brazil |
| Walker (2018) | Transmissibility of emerging viral zoonoses |
| Wang (2019) | Evolutionary history of Simbu serogroup orthobunyaviruses in the Australian episystem |
| Wernike (2016) | Effective interference between Simbu serogroup orthobunyaviruses in mammalian cells |
| Wirth (1989) | The neotropical biting midges related to *Culicoides paraensis* (diptera, ceratopogonidae) |
| **Wrong publication type (n=61)** | |
| Anderer (2024) | Oropouche Virus Spreads to New Regions in Latin America |
| Anon (2016) | Existing drugs could fight lethal viral infections A1 - Anonymous |
| Anon (2024) | Oropouche virus in Europe: Spread is unlikely |
| Barreto (2024) | Environmental Crisis and the Emergence of the Oropouche: A Potential Public Health Problem |
| Castro (2024) | Unprecedented spread and genetic evolution of the Oropouche virus: Infectious diseases |
| CDC (2025) | Countries and Territories with Recent or Previous Oropouche Virus Transmission |
| Corrales (2021) | Seroprevalence against virus and zoonotic parasitic diseases in sloths of western Panama |
| Del Valle-Mendoza (2017) | An outbreak of febrile syndromes in the north of Peru: Emerging and reemerging arboviruses |
| do Rosario (2024) | Emerging threat of Oropouche virus in Brazil: an urgent call for enhanced surveillance and response |
| Downs (1962) | Virus studies in Trinidadian swamps |
| Downs (1975) | Malaria: the great umbrella |
| Drexler (2025) | Oropouche virus more widespread than thought |
| Fabbri (2006) | Evidence of a new genotype of Oropouche virus (OROV) in Argentina, 2005 |
| Feldmann (2011) | Truly emerging--a new disease caused by a novel virus |
| Figueiredo (2011) | Molecular diagnosis of viral infections: Arboviruses and rodent-borne viruses |
| Fujita (2024) | Oropouche in Brazil in 2024 |
| Gomez (2019) | First report of the oropouche virus in Colombia |
| González (2015) | Oropuche virus: A virus present but ignored |
| Gopalsamy (2024) | A View on the Emerging Concern of Oropouche Fever in Brazil and Its Diagnosis |
| Guevara (2015) | First evidence of una virus infections in Indigenous and non-Indigenous communities in Loreto, Peru |
| Henry (2018) | Etymologia: Oropouche [oro-pooche] virus |
| Jurado-Cobena (2025) | Efficient Expression of Oropouche Virus Nonstructural Proteins NSs and NSm |
| Khan (2015) | The role of SARM1 in Toll-like receptor and viral-induced neuronal apoptosis (INM7P.347) |
| Laine (2025) | Emerging and Resurging Vector-Borne Illnesses |
| LeDuc (1989) | Oropouche fever |
| Lenharo (2024) | Mysterious Oropouche virus is spreading: what you should know |
| Marcio (2012) | Full length sequencing and genome assembling strategies of distinct arboviruses using pyrosequencing data |
| Martins-Luna (2018) | OROV isolation in a northern region of Peru: First molecular identification and clinical characteristics |
| Moutinho (2024) | Little-known virus surging in Latin America may harm fetuses |
| Moutinho (2024) | Little-known virus is on the rise in South America |
| Nielsen-Saines (2024) | Oropouche virus and potential birth defects |
| Noji (1994) | Emergency department response to a disaster from an emerging pathogen |
| Nowotny (2008) | Emergence of mosquito-borne Bunya-, toga-, and reoviruses in central Europe |
| O'Laughlin (2025) | Oropouche Virus: A Rising Threat in the Western Hemisphere |
| Onyango (2014) | De novo micro satellite marker mining from scarce amounts of *Culicoides* genomic DNA: Pathway to understanding dispersal and population of the vector of oropouche virus |
| Organización Panamericana de la Salud (2003) | Zoonosis y enfermedades transmisibles comunes al hombre y a los animales: parasitosis, v.3, 3 ed |
| PAHO (1962) | Research and research needs in arthropod-borne viruses diseases in Latin America |
| Perú Instituto Nacional de Salud (1997) | Curso Internacional Enfermedades Virales Emergentes |
| Quaglia (2024) | Clues emerge about an obscure virus' sudden spread |
| Rio (2025) | Boletim Educação em Debate - Ed. 14 |
| Rodriguez-Morales (2024) | Re-emergence of Oropouche virus in Brazil and Latin America |
| Romero-Alvarez (2018) | Emergent viruses in America: The case of Oropouche virus |
| Sah (2024) | Oropouche fever fatalities and vertical transmission in South America: implications of a potential new mode of transmission |
| São (2024) | Cuidados em relação ao recém-nascido com suspeita ou confirmação de transmissão vertical pelo OROV: epidemiologia |
| Schweitzer (2025) | Little-Known Oropouche Virus Is Spreading: What Clinicians Need to Know About the Insect-Borne Illness |
| Secretaria da Saúde do Estado de São Paulo (2024) | Vigilância da febre do oropouche no Estado de São Paulo |
| Silva-Caso (2017) | Oropuche virus identification as an emerging etiological agent responsible for acute febrile disease in a eastern middle region of the Peruvian jungle |
| Silva-Caso (2018) | First outbreak of Oropouche Fever reported in a non-endemic central-western region of the Peruvian Amazon. Molecular diagnosis and clinical characteristics |
| Soane (2019) | Novel screening ELISA for sensitive detection of Mayaro virus infected patients |
| Tauro (2018) | An outbreak of chikungunya: Tracking the epidemic threat in Salvador, Brazil |
| Taylor (2024) | PAHO: Americas report record dengue and Oropouche cases |
| Taylor (2024) | Oropouche fever: Latin America on high alert for virus that can cause stillbirths |
| Taylor (2024) | Why are doctors being warned about the Oropouche virus? |
| Travassos (1989) | Arbovirus research in the Brazilian Amazon |
| Valero (2017) | Oropouche Virus: what is it and how it is transmitted? |
| Vasconcelos (1998) | Arboviruses Pathogenic for man in Brazil |
| Vasconcelos (2010) | Oropouche fever virus: Molecular epidemiology and evolution |
| Veggiani (2019) | Monitoring of *Culicoides* (Ceratopogonidae), parasite and arboviral vectors in intervened forest environments, Tucuman-Argentina |
| Vidal-Cardenas (2013) | Exploration malaria and other vector-borne illnesses incidence in entomological workers |
| Webber (2021) | Oropouche virus in vitro replication is inhibited by acridone |
| WHO (2024) | WHO launches global strategic plan to fight rising dengue and other Aedes-borne arboviral diseases |
| **Duplicate reference (n=51)** | |
| Alvarez-Falconi (2010) | Oropuche fever outbreak in Bagazan, San Martin, Peru: epidemiological evaluation, gastrointestinal and hemorrhagic manifestations |
| Anderson (1965) | Oropouche virus: a new human disease agent from Trinidad, West Indies |
| Araiuo (1978) | Ultra-structural hepatic alterations in hamsters experimentally inoculated with the arbovirus Oropouche (type BeAn 19991) |
| Barbiero (2025) | Persistent Oropouche virus viremia in two travellers returned to Italy from Cuba, July 2024 |
| Borborema (1982) | Description of the first outbreaks of Oropouche fever recognized in the state of Amazonas, Brazil |
| Borborema (1982) | Primeiro registro de epidemias causadas pelo virus Oropouche no Estado do Amazonas |
| Cardoso (2010) | Novos registros e potencial epidemiológico de algumas espécies de mosquitos (Diptera, Culicidae), no Estado do Rio Grande do Sul |
| Casseb (2015) | Prevalência de anticorpos contra arbovírus da família bunyaviridae em búfalos de água |
| Castilletti (2024) | Oropouche virus risk for European travellers to Cuba: an emerging public health concern |
| Cruz (2009) | Serological survey for arboviruses in Juruti, Para State, Brazil |
| Cruz (2025) | Co-Circulation of 2 during Outbreak, Amazon Region of Peru, 2023-2024 |
| de Figueiredo (2004) | Doenças exantemáticas e primeira epidemia de dengue ocorrida em Manaus, Amazonas, no período de 1998-1999 |
| Dixon (1981) | A multidisciplinary programme of infectious disease surveillance along the Transamazon Highway in Brazil. II. Epidemiology of arbovirus infections |
| Dixon (1981) | Programa multidisciplinário de vigilancia de las infermedades infecciosas en zonas colindantes con la Carretera Transamazónica en Brasil. II. Epidemiología de las infecciones por arbovirus |
| Freitas (1980) | Epidemic of Oropouche virus in the eastern part of the State of Para, 1979 |
| Garcia (2016) | Detection of Oropouche viral circulation in Madre de Dios region, Peru (December 2015 to January 2016) |
| Graf (2025) | Expansion of Oropouche virus in non-endemic Brazilian regions: analysis of genomic characterisation and ecological drivers |
| Hoch (1987) | Criaderos de *Culicoides paraensis* y opciones para combatirlos mediante el ordenamiento del medio |
| Hoch (1987) | El virus Oropouche. Transmisión en el laboratorio por *Culex Quinquefasciatus* |
| Hoch (1987) | Oropouche virus. Transmission in the laboratory by *Culex quinquefasciatus* |
| Hoch (1987) | El virus Oropouche: transmision en el laboratório por *Culex quinquefasciatus* |
| Hoch (1987) | Criaderos de *Culicoides paraensis* y opciones para combatirlos mediante el ordenamiento del medio |
| Hoch (1987) | Laboratory transmission of Oropouche virus by Culex quinquefasciatus say |
| Izurieta (2009) | Assessing yellow Fever risk in the Ecuadorian Amazon |
| Laroque (2014) | Epidemiologic survey for arbovirus in Galician capuchin monkeys (*Cebus flavius*) free living in Paraiba and captive capuchin monkey (Cebus libidinosus) from Northeast Brazil |
| LeDuc (1981) | Epidemic Oropouche virus disease in northern Brazil |
| LeDuc (1982) | Enfermedades epidémicas causadas por el virus Oropouche en el norte de Brasil |
| LeDuc (1982) | Epidemic illness caused by Oropouche virus in northern Brazil |
| LeDuc (1982) | Enfermedades epidemicas causadas por el virus Oropouche en el norte de Brasil |
| Maier (2025) | Imported Oropouche fever to Germany in a returning traveller from Cuba |
| Nunes (2009) | Eco-epidemiologia dos arbovírus na área de influência da rodovia Cuiabá-Santarém (BR 163), Estado do Pará, Brasil |
| Organização Pan-Americana da Saude (2024) | Orientações provisórias de vigilância entomológica e medidas de prevenção contra vetores do vírus Oropouche |
| Organización Panamericana de la Salud (1982) | Boletín Epidemiológico, v. 3, n. 5, Oct. 1982 |
| Organización Panamericana de la Salud (1982) | Fiebre de oropouche en Brasil |
| Organización Panamericana de la Salud (2022) | Recomendaciones para la detección y el diagnóstico por laboratorio de infecciones por arbovirus en la Región de las Américas |
| Organización Panamericana de la Salud (2024) | Orientaciones provisionales para la vigilancia entomológica y las medidas de prevención de los vectores del virus de Oropouche |
| Pan American Health Organization (PAHO) (1982) | Oropouche fever in Brazil |
| PAHO (1973) | Surveillance and research on infectious diseases along the trans-Amazon highway |
| PAHO (1982) | Epidemiological Bulletin, v. 3, n.5, Oct. 1982 |
| PAHO (1982) | Fiebre de Oropouche en Brasil |
| PAHO (2024) | Orientations provisoires pour la surveillance entomologique et les mesures de prévention concernant les vecteurs du virus Oropouche |
| PAHO (2025) | Interim guidance on entomological surveillance and prevention measures for Oropouche virus vectors |
| PAHO (2025) | Epidemiological Update Oropouche in the Americas Region |
| Pinheiro (1974) | Vigilancia e investigación de las enfermedades infecciosas a lo largo de la carretera transamazónica del Brasil |
| Pinheiro (1982) | Meningitis associated with Oropouche virus infections |
| Pinheiro (1964) | An epidemic of Oropouche virus in Belem. (Preliminary note) |
| Rosa (1996) | Outbreak of oropouche virus fever in Serra Pelada, municipality of Curionopolis, Para, 1994 |
| Schwarz (2022) | Oropouche orthobunyavirus infection is mediated by the cellular host factor Lrp1 |
| Vasconcelos (1989) | First report of epidemics caused by Oropouche virus in Maranhao and Goias States, Brazil |
| Vasconcelos (1989) | 1st register of an epidemic caused by Oropouche virus in the states of Maranhao and Goias, Brazil |
| Vasconcelos (1989) | Epidemic outbreaks of Oropouche virus in the Maranhão and Goias states, Brazil |
| **Preprint duplicate reference (n=12)** | |
| Azevedo (2024) | Genomic and phenotypic characterization of an Oropouche virus strain implicated in the 2023-24 large-scale outbreak in Brazil |
| Barbosa (2020) | Oropouche virus glycoprotein topology and cellular requirements for virus assembly |
| Delatorre (2024) | Oropouche virus outbreak in southeast, Brazil: expanding beyond the Amazonian endemic region |
| de Melo Iani (2024) | Rapid viral expansion beyond the Amazon basin: increased epidemic activity of Oropouche virus across the Americas |
| do Nascimento (2019) | Oropouche virus detection in saliva and urine |
| Gutierrez (2019) | The evolutionary dynamics of Oropouche Virus (OROV) in South America |
| Naveca (2024) | Emergence of a novel reassortant Oropouche virus drives persistent human outbreaks in the Brazilian Amazon region from 2022 to 2024 |
| Scachetti (2024) | Reemergence of Oropouche virus between 2023 and 2024 in Brazil |
| Stubbs (2021) | Vesicular stomatitis virus chimeras expressing the Oropouche virus glycoproteins elicit protective immune responses in mice |
| Usuga (2024) | Co-circulation of two lineages of Oropouche virus in the Amazon basin, Colombia, 2024 |
| Walsh (2024) | Short Report: Investigating Oropouche as a possible etiology for fevers of unknown origin in a clinical cohort from Colombia, 2013-2015 |
| Wise (2019) | Oropouche virus cases identified in Ecuador using an optimised rRT-PCR informed by metagenomic sequencing |

1. Peters MDJ, Marnie C, Tricco AC, et al. Updated methodological guidance for the conduct of scoping reviews. JBI Evid Synth **2020**; 18:2119–26.
